# A review of tunable photonics: Optically active materials and applications from visible to terahertz

**DOI:** 10.1016/j.isci.2022.104727

**Published:** 2022-07-05

**Authors:** Joo Hwan Ko, Young Jin Yoo, Yubin Lee, Hyeon-Ho Jeong, Young Min Song

**Affiliations:** 1 School of Electrical Engineering and Computer Science, Gwangju Institute of Science and Technology, Gwangju 61005, Republic of Korea; 2 School of Materials Science and Engineering, Gwangju Institute of Science and Technology, Gwangju 61005, Republic of Korea; 3 Anti-Viral Research Center, Gwangju Institute of Science and Technology, Gwangju 61005, Republic of Korea; 4 AI Graduate School, Gwangju Institute of Science and Technology, Gwangju 61005, Republic of Korea

**Keywords:** Photonics, Applied physics, Materials science

## Abstract

The next frontier of photonics is evolving into reconfigurable platforms with tunable functions to realize the ubiquitous application. The dynamic control of optical properties of photonics is highly desirable for a plethora of applications, including optical communication, dynamic display, self-adaptive photonics, and multi-spectral camouflage. Recently, to meet the dynamic response over broad optical bands, optically active materials have been integrated with the diverse photonic platforms, typically in the dimension of micro/nanometer scales. Here, we review recent advances in tunable photonics with controlling optical properties from visible to terahertz (THz) spectral range. We propose guidelines for designing tunable photonics in conjunction with optically active materials, inherent in wavelength characteristics. In particular, we devote our review to their potential uses for five different applications: structural coloration, metasurface for flat optics, photonic memory, thermal radiation, and terahertz plasmonics. Finally, we conclude with an outlook on the challenges and prospects of tunable photonics.

## Graphical abstract

**Figure:**
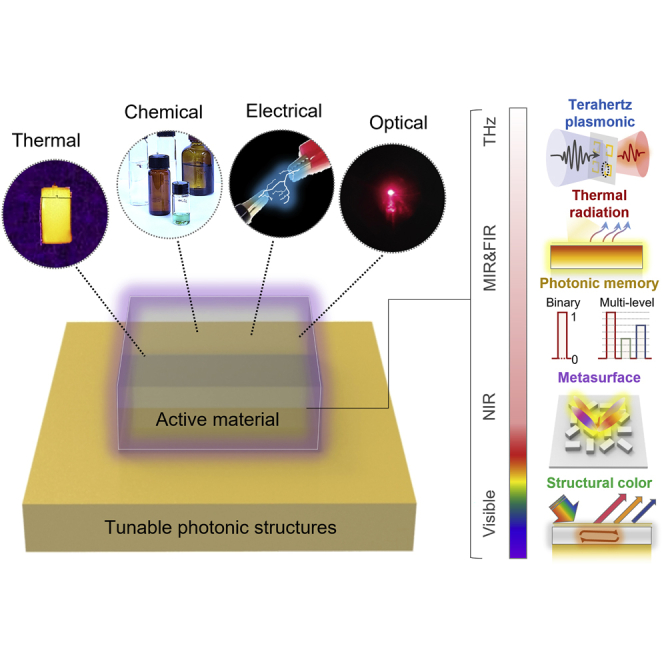

## Introduction

The dream of developing an artificially addressable optical system has come closer to execution in recent decades, thanks to the conspicuous progress of fundamental physics and manufacturing technology. After the appearance of the *Lycurgus Cup* shows peculiar dichroic/multifarious properties in ancient Roman (Freestone et al., 2007), humankind has shown the capability to create diverse optical responses that could perform complex light manipulation with a prominent variation. Since Dennis Gabor proposed holography (Gabor, 1972), various media and films depict a future active-video technology, and eventually, tunable photonics are currently in realization (Fang et al., 2020; Han et al., 2019; Ren et al., 2020; Wuttig et al., 2017). Future headway over the predicted areas sensitively relies on the availability of improved physics in light-matter interaction and/or accompanied active materials. Therefore, before actively addressing the optical response, understanding the interaction between photonic structures and light unravels design rules for the next generation of the reconfigurable optical system.

After the first formulation of photons and their interaction with an electron, by Paul Dirac in 1927 (Dirac, 1927) and re-formulation by Enrico Fermi in 1932 (Fermi, 1932), light-matter interactions are fundamentally considered as not only an electromagnetic wave propagating at the speed of light in a vacuum but also photons coupling to material quasiparticles (plasmons, phonons, and excitons) (Rivera and Kaminer, 2020). Following the progress in understanding the fundamental interaction of light, many developments bring the generalized view of the electromagnetic waves and photons at the core of every light-matter interaction. As well known, when the light waves encounter a photonic structure, electromagnetic waves are either transmitted, reflected, absorbed, refracted, polarized, diffracted, or scattered depending on the structure/material of the object and the wavelength of the light. Meanwhile, interaction between photon and material quasiparticles includes Bloch photon, cavity photon, surface polaritons, and bulk plasmons, which produce optical resonance and/or photonic bandgap. (Rivera and Kaminer, 2020). Based on the interaction between light and matters, various photonic structures have been designed for efficiently manipulating the incident light to target response. Representatively, in this review, we will cover several types of structures including photonic crystals, which create a photonic bandgap from periodic structure (Zhang et al., 2015), plasmonics based on the coupled plasmon-polaritons (Ozbay, 2006), and resonance cavity, enabling light confinement in a reflective cavity (Wang et al., 2020). Furthermore, optical waveguides have a crucial role in low-loss light propagation (Selvaraja and Sethi, 2018). Typically, metasurfaces show great controllability of amplitude, phase, polarization, and refractive indices (Yu and Capasso, 2014). Recent advances of innovatively progressed photonic structures offer feasible opportunities to realize optical response in many applications. Meanwhile, scaling down the light-matter interaction in a shorter wavelength range still remains a laborious task. For that reason, the realization of integrating photonic structures with the diverse platforms in the range of nano- to micro-dimension is a key challenge for enlarging the operating spectral range (*e.g.*, from visible to terahertz (THz) frequency).

The next step toward a dynamic optical response is to escape from the static state, which is fixed by initial structures. One class of active materials that have shown considerable attention for tunable photonic application is phase change material (PCM) due to their large variations in the optical properties in both imaginary and real parts of the complex refractive index. The PCMs’ large variations in optical properties and great compatibility with complementary metal-oxide semiconductor (CMOS) (*e.g.* Ge-Sb-Te (GST), VO_2_) facilitate the realization of highly compact switchable photonic applications including metasurface, neuromorphic photonic memory device, and terahertz photonic devices, over the broad spectral range (Abdollahramezani et al., 2020; Che et al., 2020; Jeong et al., 2020; Neubrech et al., 2020; Shastri et al., 2021; Xu et al., 2022). Their optical modulation stems from the changes in their atomic bonding configuration according to metal-insulator transition (MIT). The introduction of free carriers in metallic state results in excessive loss due to free carrier absorption (FCA), especially around visible range. In some cases, this extinction coefficient has been utilized for non-trivial phase shift for ultrathin colorations (Bakan et al., 2016). Also, C. Ríos et al. show a drastic color variation based on simple film configuration (Ríos et al., 2016). When switching their material phase from amorphous to crystalline state, the associated refractive index (real part) changes from high to low while changing the extinction coefficient (imaginary part) from low to high, leading to clearly blue-shifted color modulation. However, the contemporaneous increase of extinction coefficient (*i.e.*, optical loss) fundamentally limits the range of optical wavelength regime, thus many optical applications. Recent progress in the technical development unlocks such limits, *e.g.*, by searching and developing new material composition or introducing dopant into the known active materials (Yang et al., 2021; Zhang et al., 2019). Aside from PCMs, diverse optically active materials have been emerged for manipulating the photonic responses with appropriate complex refractive index and tuning range. The active materials include 1) thermal energy-driven phase-change/-transition materials, 2) chemical/electrochemical-reaction-based refractive index tuning in conducting polymers, dielectric, or metals, 3) electrical energy-driven dynamic birefringence material (*i.e.*, LCs), and 4) electrical/optical stimulation for free-carrier pumping in dielectric or semiconductor, resulting in modulation of the complex refractive index.

In this review, we focus on the diverse optically active materials in regard to their physical mechanism for tuning, operating frequency (*i.e.*, target wavelength), applications, and accompanied advantages and limitations. We begin with the introduction of optically tunable materials and their mechanisms. Then, we discuss the categorized potential applications following each optical band including visible, near-infrared (NIR), mid-infrared (MIR), far-infrared (FIR), and THz frequency. The discussion of categories covers the characteristic of spectral range and the recently developed applications with the operating stability, switching speed, spatial range, and structural uniformity/scalability, and in some cases, chromaticity, polarization, or thermal properties.

## Optically active materials and light-matter interactions

**Figure 1:**
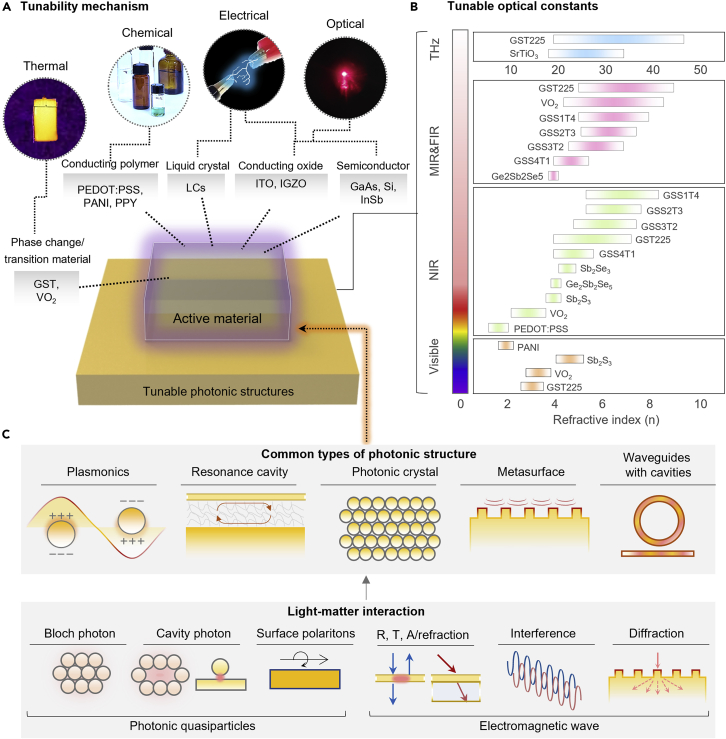
Operation mechanisms of tunable photonics and refractive index variation (A) Tunable photonics with optically active material and key operation mechanisms. (B) Refractive indices variation comparison of optically active materials corresponding to each spectral range. (C) Schematic of common types of photonic structures and light-matter interaction divided in photonic quasiparticles and electromagnetic waves, respectively. R, T, and A represent reflection, transmission, and absorption, respectively.

In this section, we briefly introduce the optically active materials with common types of photonic structures and associated light-matter interactions. Depending on each type of optically active materials used in the photonic structures, representative external stimuli are presented in Figure 1A, including thermal, chemical, electrical, and optical methods. Based on these categories, we introduce the representative active materials with their physical mechanism and related issues, including each operating spectral range, the application, and the accompanied advantages and limitations (Table 1). In order to provide an innate property in terms of tuning functions, we compared refractive indices and its tuning range corresponding to the materials and spectral range (Figure 1B and Table 2).

**Table 1:** Comparison of modulation methods corresponding to operation elements

Stimuli	Example	Physical mechanism	Speed	Advantages	Limitations	Operating wavelength	Ref.
Thermal	GST	Phase change	500 ps	Reversible Stable	Energy loss Diffusion effect	Visible to THz	(Zhang et al., 2019, 2021)
	VO_2_	Phase transition	1.3 ms	Reversible	Energy loss	Visible to THz	(Wu and Liou, 2011)
Chemical/electrochemical	Polyaniline	Redox reaction	32 ms	Low power consumption	Modulation speed Need electrolyte	Visible to FIR	(Peng et al., 2019)
	PEDOT	Redox reaction	9 ms	Low power consumption	Modulation speed Need electrolyte	Visible to THz	(Karst et al., 2021; Liu et al., 2019a; Wang et al., 2018)
	Mg/Pd	Hydrogenation	∼100 s	Great sensitivity Low cost	Operation speed	Visible to NIR	(Li et al., 2018a)
	Graphene	Fermi level tuning	∼1 s	Low power consumption Scalable	Modulation speed Need electrolyte	Visible to THz	(Ergoktas et al., 2021; Salihoglu et al., 2018)
Electrical	LCs	Birefringence change	5 ms	Reversible CMOS compatible	Slow response	Visible to THz	(Chien et al., 2017)
	ITO	Carrier density modulation	10 MHz	Fast switching	Energy loss	Visible to MIR	(Huang et al., 2016)
	Graphene	Carrier density modulation	1 GHz	Low power consumption	Hard to array configuration	NIR to THz	(Zeng et al., 2018)
Optical	Si	Carrier density modulation	20 ps	Fast switching	Energy loss	Visible to THz	(Cai et al., 2018)
	GaAs	Carrier density modulation	6 ps	Fast switching	Energy loss	MIR to THz	(Shcherbakov et al., 2017)

**Table 2:** Comparison of refractive index variation corresponding to the spectral range

Spectral range	Material	Reference wavelength	Refractive index (*n*)			Reference
			Max.	Min.	*Δn*	
Visible	GST-225	0.532 μm	2.8	1.9	0.9	(Zheng et al., 2018)
	VO_2_	0.5 μm	3.1	2.1	1.0	(Van Bilzen et al., 2015)
	Sb_2_S_3_	0.5 μm	4.4	3.3	1.1	(Liu et al., 2020)
	Polyaniline	0.532 μm	1.6	1.0	0.6	(Barbero and Kötz, 1994)
NIR	PEDOT:PSS	1.6 μm	1.4	0.6	0.8	(Karst et al., 2021)
	VO_2_	2 μm	2.9	1.5	1.4	(Derkaoui et al., 2019)
	Sb_2_S_3_	1.5 μm	3.5	2.9	0.6	(Delaney et al., 2020)
	Ge_2_Sb_2_Se_5_	2 μm	3.5	3.1	0.4	(Zhang et al., 2019)
	Sb_2_Se_3_	1.55 μm	4.1	3.4	0.7	(Delaney et al., 2020)
	GSST-2241	2 μm	4.8	3.2	1.6	(Zhang et al., 2019)
	GST-225	1.5 μm	6.3	3.2	3.1	(Julian et al., 2020)
	GSST-2232	2 μm	6.5	4	2.5	(Zhang et al., 2019)
	GSST-2223	2 μm	6.7	4.5	2.2	(Zhang et al., 2019)
	GSST-2214	2 μm	7.4	4.5	2.9	(Zhang et al., 2019)
MIR & FIR	Ge_2_Sb_2_Se_5_	15 μm	3.4	3	0.4	(Zhang et al., 2019)
	GSST-2241	15 μm	4.6	3.2	1.4	(Zhang et al., 2019)
	GSST-2232	15 μm	6	3.8	2.2	(Zhang et al., 2019)
	GSST-2223	15 μm	6.5	4.3	2.2	(Zhang et al., 2019)
	GSST-2214	15 μm	7	4.2	2.8	(Zhang et al., 2019)
	VO_2_	5 μm	7.6	3.6	4	(Tian and Li, 2018)
	GST-225	15 μm	8	4.2	3.8	(Julian et al., 2020)
Terahertz	SrTiO_3_	0.2 THz	30	15	15	(Singh et al., 2011)
	GST-225	1 THz	42	16	26	(Makino et al., 2019)

### Thermal energy-induced phase-change/-transition materials

PCMs are materials that exhibit excellent changes in optical property upon a solid-state phase transition. Because of their nonvolatile, quick, reversible, and physically distinct phase changes between amorphous and crystalline state, chalcogenide alloys, *i.e.*, GST and its family materials have been utilized in applications in both optical and resistive memory devices (Hempelmann et al., 2022). They undergo a change in atomic bonding configuration at two different temperature regimes: 1) crystallization temperature, at which amorphous phase crystallizes (critical temperature, *T*_*c*_ ≈ 160°C), and 2) melting temperature, at which crystalline phase becomes disordered (melting temperature, *T*_*m*_ ≈ 600°C). However, due to the increased excessive loss in metallic state of GST, the large extinction coefficient is problematic for many optical applications. As an alternative, Ge–Sb–Se–Te (GSST), recently, have been suggested with its broadband optical transparency with many kinds of alloys including Ge_2_Sb_2_Te_5_ (GST-225), Ge_3_Sb_2_Te_6_ (GST-326), Ge_2_Sb_2_Se_1_Te_4_ (GSST-2214), Ge_2_Sb_2_Se_2_Te_3_ (GSST-2223), Ge_2_Sb_2_Se_3_Te_2_ (GSST-2232), Ge_2_Sb_2_Se_4_Te_1_ (GSST-2241), and Ge_2_Sb_2_Se_5_ (Zhang et al., 2019). Among the transition metal oxides, VO_2_ has been widely investigated due to the drastic MIT near room temperature (critical temperature, *T*_*c*_ ≈ 70°C) with volatile property. The phase transition causes change in an electrical conductivity of three to four orders of magnitude, corresponding a lattice structure switching between a monoclinic (insulating phase) and a tetragonal (metallic phase) state. However, as indicated in Table 1, PCM and transition metal oxides not only require the large energy consumption originated from the driving conditions for phase transition but also intrinsically possess the large optical losses in certain wavelength regimes, which should be considered for the device designs.

### Chemical/electrochemical reaction-based active materials

One of the representative optical modulations based on a chemical reaction is hydrogenation/dehydrogenation kinetics such as Mg and Pd. Mg can transit its phase from metal to dielectric with forming magnesium hydride (MgH_2_) upon hydrogen loading, causing its excellent optical modulation. For example, X. Duan et al. represented tunable nanoplasmonic system for the switchable structural color with the configuration of Mg/Ti/Pd (Duan et al., 2016). Despite its great tunability, the hydrogenation process for switching the plasmonic modes usually needs nearly 600 s and the recovery process needs about 2000 s. In contrast, organic electrochromic materials have advantages such as simple manufacturing, low power consumption, cost effectiveness, and flexibility. The representative conducting polymers include polyaniline (PANI), polypyrrole (PPy), and poly (3,4-ethylene dioxythiophene) (PEDOT). By electrochemically switching the redox states via electrochemical reaction and altering the molecular polarizability of such polymers, they reversibly shift extinction spectrum. Meanwhile, inorganic electrochromic materials (*e.g.*, TiO_2_, WO_3,_ NiO, and V_2_O_5_) perform better cycling durability and chemically stability (Ma and Wang, 2017; Tong et al., 2016). For example, J. Eaves-Rathert et al. represented electrochemically actuated TiO_2_ metasurface (Eaves-Rathert et al., 2022). Upon Li ion intercalation, the tetragonal symmetry of anatase is broken, forming orthorhombic Li_0.5_TiO_2_ with optical property change. Also, the Fermi level of graphene can be modulated through electrostatic gating or chemical doping. O. Salihoglu et al. showed a dynamic infrared emission tuning with ionic liquid intercalates into graphene layers, enabling increase of charge density of graphene, which suppress the IR absorption (Salihoglu et al., 2018). Despite the stable and reversible variation of these electrochromic organic/inorganics, they still suffer from the limited modulation speed and requirements of external driving conditions such as electrolytes.

### Electrical energy-driven birefringence material

LCs, which exist between a liquid and the crystalline phase, are a state of matter characterized by a partial or complete degree of positional disorder in crystalline solid. LCs exist in various phases, namely, isotropic, nematic, and smectic (Stevenson et al., 2005). Isotropic LCs possess random orientations, whereas nematic LCs show fixed orientations, and smectic LCs have fixed orientations with well-defined planes. The large optical birefringence of LCs, which can be controlled by thermal, chemical, or electrical stimulation, enables an obvious potential for a tunable optical response. Owing to the feasibility of integration process of LCs with photonic structures, it also can be exploited as the tunable photonics. After the first report of integration of LCs with the split ring resonators in microwave range (resonance frequency = 11.08 GHz), photonic structures have been integrated with LCs over the broad spectrum from visible to terahertz range (Zhao et al., 2007). It is noteworthy that CMOS technology could be a cornerstone for the mass production of dynamic photonics based on the highly mature LCD technology. For example, Lumotive Inc. produces an ultra-compact light detection and ranging (LiDAR) system based on LC through conventional 0.13 μm CMOS semiconductor foundry with a Himax Inc. semiconductor experts (Kim et al., 2021c; Mikheeva et al., 2022). However, they have some limitations to be resolved including the slow modulation speed (microsecond level) and large structural volume.

### Electrical/optical stimulation induced free-carrier pumping materials

In case of optical property tuning via free-carrier density modulation, the medium immediately changes the refractive index through electric-/optical energy-driven carrier pumping. According to the Drude model, refractive index of semiconductors can be tuned by altering their carrier densities and effective masses (Gu et al., 2012; Shcherbakov et al., 2017). Likewise, conductive oxides can also be heavily doped so that the plasma frequency reaches the NIR regime (Feigenbaum et al., 2010; Noginov et al., 2011). As a result, semiconductors, and conductive oxides, such as indium tin oxide (ITO), indium gallium zinc oxide (IGZO), GaAs, Si, and InSb, are optically tunable by modulating their free carrier density through electrical or optical pumping. Electrical/optical pumping mechanism can be achieved in fast speed up to GHz level in modulation speed. Meanwhile, they also have some limitations such as narrow modulation range and high energy loss. For example, in case of GaAs and InSb, the maximum carrier densities are on the order of 10^18^ cm^−3^, limiting the refractive index modulation only at MIR and THz frequencies (Cui et al., 2019).

### Photonic structures and light-matter interaction

Optically active materials are integrated on various photonic structures, allowing light-matter interactions to control optical responses without structural reconstruction. Figure 1C represents common types of photonic structures and basis mechanisms. The interaction between light and photonics is classified into photonic quasiparticle and electromagnetic waves. Photonic quasiparticles include Bloch photons (*i.e.*, effective localization of light interfered with periodic structures), cavity photons (*i.e.*, light confinement in a reflective cavity and quasiparticles), and surface polaritons (*i.e.*, light confinement by coupling between plasmons and polaritons). Moreover, bulk plasmons and localized plasmons, and even acoustic phonons are special examples of photonic quasiparticles (Rivera and Kaminer, 2020). Light as an electromagnetic wave interacts with photonics in the way of transmission, reflection, absorption, refraction, polarization, diffraction, or scattering depending on the optical constant or structure of photonics and the wavelength of the light. Also, each case of photonic structure shows inherent optical responses such as manipulating the resonance with strong absorption, photonic bandgap, and wavefront shaping. We present five common types of photonic structure including 1) plasmonics, which cause strong light absorption based on light confinement coupled by plasmon-polaritons, and 2) resonance cavity, which shows a strong light confinement in a cavity space. Moreover, 3) photonic crystals create a photonic bandgap based on Bloch photons, enabling distinct selective reflection, and 4) metasurfaces show great controllability of amplitude, phase, and polarization, and spatially varying refractive indices, allowing the manipulation of light (Yu and Capasso, 2014). Finally, 5) optical waveguides with resonant cavities have a capability in low loss propagation based on efficient coupling to diverse cavity mechanisms.

## Spectral range and key applications

**Figure 2:**
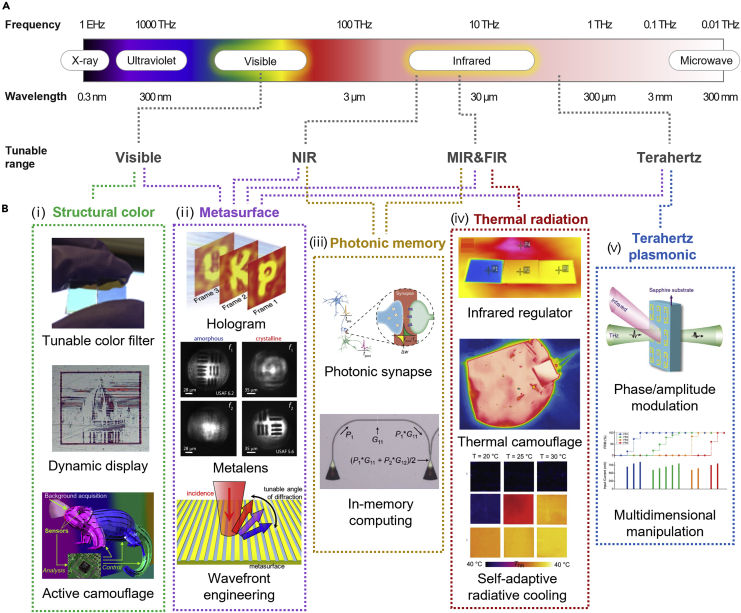
Overview of tunable photonics from visible to THz (A) Frequency and wavelength range and corresponding spectral range from visible to THz. (B) Representative tunable photonics applications corresponding to each spectral range. (ⅰ) Structural color using visible spectral range including tunable color filter, Reproduced with permission from (He et al., 2020), Copyright@2020 The Optical Society, dynamic display, Reproduced with permission from (Hosseini et al., 2014), Copyright@2014 Springer Nature, and adaptive camouflage, Reproduced with permission from (Wang et al., 2016a), Copyright@2016 American Chemical Society. (ⅱ) Metasurface for flat optics based on all of the represented spectral range (*i.e.*, visible to THz) showing the applications of hologram, Reproduced with permission from (Li et al., 2017), Copyright@2017 Springer Nature, metalens, Reproduced with permission from (Shalaginov et al., 2021), Copyright@2021 Springer Nature, and wavefront engineering, Reproduced with permission from (Huang et al., 2016), Copyright@2016 American Chemical Society. (ⅲ) Photonic memory based on NIR and MIR including applications of photonic synapse, Reproduced with permission from (Cheng et al., 2017), Copyright@2017 AAAS and in-memory computing, Reproduced with permission from and (Ríos et al., 2019), Copyright@2019 AAAS. (ⅳ) Thermal radiation based on MIR and FIR spectral range showing applications in infrared regulator, Reproduced with permission from (Gu et al., 2022), Copyright@2022 American Chemical Society, thermal camouflage, Reproduced with permission from (Qu et al., 2018). Copyright@2018 Springer Nature, and self-adaptive radiative cooling, Reproduced with permission from (Tang et al., 2021), Copyright@2021 AAAS. (ⅴ) Terahertz plasmonic-based applications of phase/amplitude modulator, Reproduced with permission from (Gu et al., 2012), Copyright@2012 Springer Nature, and multidimensional manipulation, Reproduced with permission from (Pitchappa et al., 2019), Copyright@2019 John Wiley and Sons.

This review highlights state-of-the-art tunable photonic structures and discusses their performance with potential applications. We consider five major sections of the optical bands according to the applications of each wavelength, *i.e.*, visible, NIR, MIR & FIR, and THz frequency (Figure 2A). We outline the classification of key categories following the representative characteristics of spectral range, *i.e.*, structural coloration, metasurface for flat optics, photonic memory, thermal radiation, and terahertz plasmonics (Figure 2B).

Firstly, the visible spectral range is electromagnetic spectrum that is visible to the human eye, which is an indispensable range to convey visible information to people. Due to its importance, recent developments on dynamic photonics using visible light lead to diverse visual applications including tunable coloration, colored display, adaptive coloration, and tunable metasurface. Especially, tunable metasurfaces show huge progress over the broad spectral range including infrared and THz spectrum owing to the universal applicability including imaging, focusing, beam steering, and information transport. Secondly, NIR is the nearest range to the visible spectrum, which has large refractive index variation in many PCMs (Zhai et al., 2018), and thus promises a photon-based fast/distinctly tunable applications including photonic memory or devices. Thirdly, in the photonic application, MIR and FIR spectral range have significantly important roles in thermal radiation-based applications including thermal imaging and heat-seeking missiles operation. Typically, at ambient temperature around ambient (∼300 K), atmospheric transmittance (transparent spectral range of Earth’s atmosphere, 8–13 μm) is consistent with the blackbody thermal radiation peak, enabling the thermal management, *e.g.*, radiative cooling. Using the dynamic response, functional applications such as actively controllable radiative cooler for heat management or tunable infrared camouflage are demonstrated. Finally, in the case of THz wave, due to its widely developed technologies in information transport and sensing application, dynamic manipulation of THz photonics leads to numerous promising applications including tunable high-speed wireless communication, security screening, and multidimensional manipulation.

## Structural color and functional applications

### Dynamic coloration over the static structural color

Structural coloration offers substantial opportunities for future applications such as nanoscale printing (Li et al., 2016), colorimetric sensor (Yoo et al., 2020a, 2022), multiplexed color display (Lee et al., 2021), and flexible display (Chung et al., 2012). Compared to the traditional dyes/pigments, structural coloration has great potential due to its fine tunability, sustainability, and durability. Several strategies for high color purities and design rules have been successfully demonstrated based on the photonic crystals, plasmonic nanostructure, and thin-film interferometer (Jung et al., 2022; Kim et al., 2019a, 2019b; Ko et al., 2022; Lee et al., 2022). The resonance wavelength and the associated plasmonic color are determined by parameters including structural dimension (Kim et al., 2020c), optical constant (Ko et al., 2020), surrounding medium (Yoo et al., 2020b), and arrangement of photonic structure (Fu et al., 2018).

**Figure 3:**
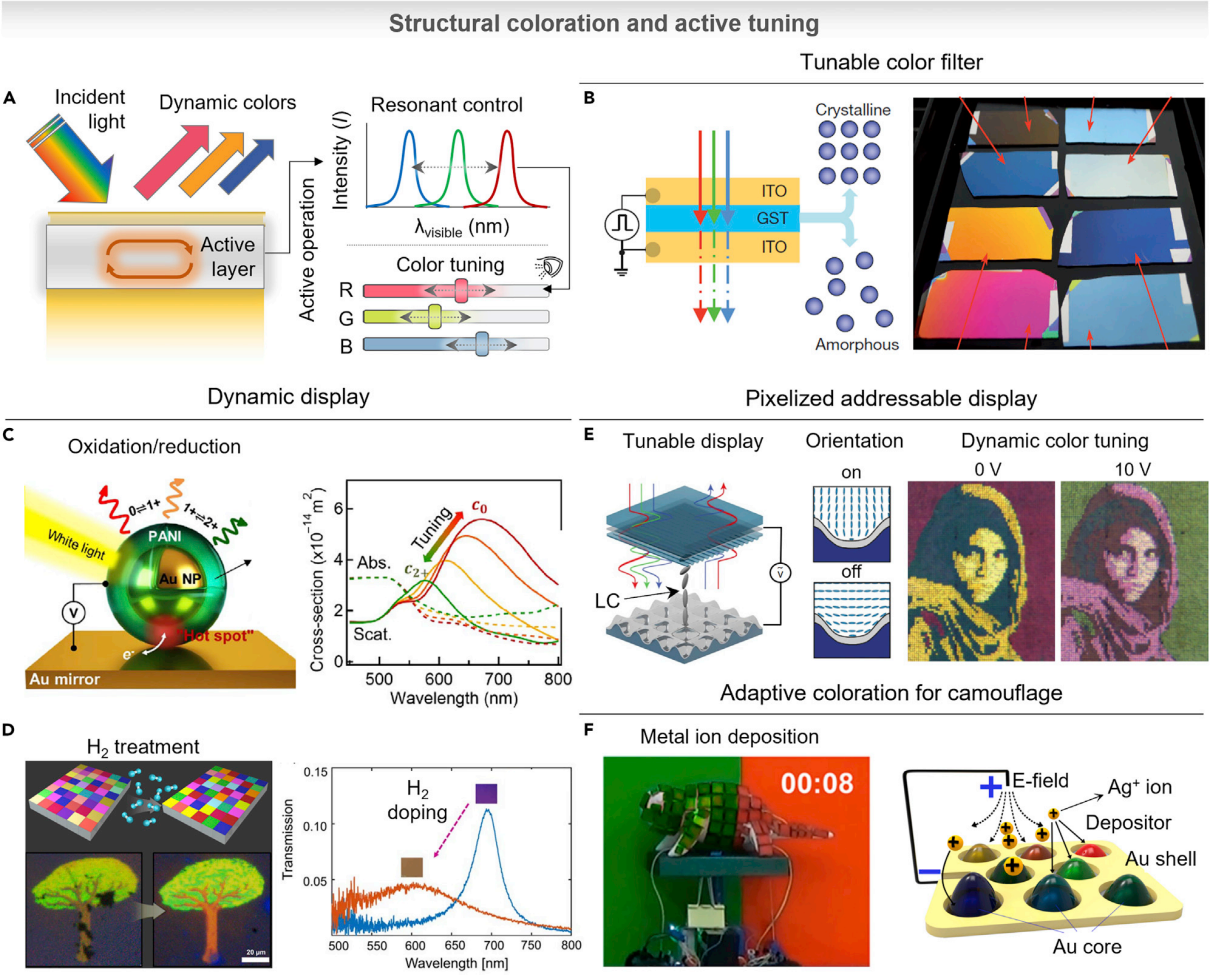
Structural coloration and dynamic functions (A) Schematic of tunable photonics with optically active materials and dynamic color change. (B) Schematic of GST-225-based tunable color filter shows a crystallinity change between the amorphous and crystalline states (left). Optical image of GST-225-based color filter (right). Reproduced with permission from (Hosseini et al., 2014), Copyright@2014 Springer Nature. (C) Dynamic color display based on polyaniline (PANI) and optical scattering (solid lines) and absorption spectra (dashed lines) for different redox states. Reproduced with permission from (Peng et al., 2019), Copyright@2019 AAAS. (D) Dynamic color display based on IGZO activated by H_2_ doping and transmittance spectra. Reproduced with permission from (Kim et al., 2020b), Copyright@2020 The Optical Society. (E) Pixelized addressable color display based on liquid crystal (LCs) with resonance shift. Reproduced with permission from (Franklin et al., 2015), Copyright@2015 Springer Nature. (F) Adaptive coloration with camouflage function based on metal ion deposition using electrodeposition/stripping method. Reproduced with permission from (Wang et al., 2016a), Copyright@2016 American Chemical Society.

Recently, over the static structural color, dynamic color tuning has been demonstrated by modulating the structure/material parameters. In this section, we summarize the advantages of different types of tunable structural colors and the following applications. Figure 3A shows the relation between the active material and the chromatic response of the photonic structure. The chromatic response has occurred by applying the functional materials into photonic structures. Although the photonic structures have static/fixed shapes, the reflected/transmitted light shows drastic color variation as a function of the change in the refractive index of optically active materials.

### Tunable color filter and resonant shift mechanism

Owing to their relatively high scattering/absorption efficiency, compared to dye/pigment-based color filters, structural color creates a much brighter color even in thinner thickness. As a basic approach, dynamic color filter presents an intuitive color variation by tuning the chromatic response. Figure 3B shows a multilayered interferometer with phase-change material (GST-225) sandwiched between two transparent oxide (ITO) layers (Hosseini et al., 2014). The phase change of GST-225 causes a large variation in the complex refractive index. Thus, the metal/ITO/GST-225/ITO structure reveals a distinct resonant color shift even in ultrathin thickness of active layer (GST-225, 7 nm). Recent approaches utilizing cavity layer (Lee et al., 2020) and metamaterial (Rui et al., 2020) with embedding an ultrathin GST absorber have enhanced color purity or tunability. For more feasible reversibility and fast transition in GST, recently, new approaches offer a modulation of the doping ratio and composition. Thus, the PCMs show lower phase-change temperature and deep operating volume (Li et al., 2022; Zhang et al., 2019). As an example, using phase-transition material (VO_2_), a multilevel color change has been demonstrated using VO_2_ and tungsten-doped VO_2_ based on multilayered Fabry-Perot cavity structure (Ko et al., 2022). Due to the different phase-transition temperature between tungsten-doped VO_2_ (W-VO_2_) and VO_2_, the resonance wavelength of cavity structure (VO_2_/SiO_2_/W-doped VO_2_) shows multilevel color variation. Furthermore, based on the electrochemical reaction, the conductive polymer (poly-thieno[3,4-b]thiophene, pT34bT) offers a dynamic and full-colored Fabry-Perot cavity under low operating voltages (Rossi et al., 2021). Even in low electrochemical potential (−1 to 0.7 V), distinct refractive index variation of pT34bT enhanced the color tunability, resulting in color modulation across the entire visible range.

### Pixelized structural color for dynamic display

The tiny volume and/or thin geometry of structural color could make an ultrahigh resolution image with small pixels, which offers possibility as a colored display. For example, Figure 3C shows a color tuning based on electrochemical redox reaction of conducting polymer (polyaniline, PANI) (Peng et al., 2019). In the redox reaction, the PANI medium shows a complex refractive index change, controlling the gap plasmons occurred between PANI-coated plasmonic gold nanoparticles and a metal surface. They showed a high operating switching rate (up to commercial video rates of 50 Hz) and great durability (over 3 months). As a practical application, the small size of this structure (<100 nm) provides small-size patterning area using aerosol jet printing method (Peng et al., 2021). This approach offers a possibility to make all-printed display with small linewidth (12.5 μm). In case of refractive index tuning by controlling doping concentration of hydrogen molecules (H_2_), Figure 3D represents a dynamic color display (Kim et al., 2020b). Using the Fabry-Perot cavity layer based on IGZO, the change of doping concentration makes different colors. The injected hydrogen atoms produce free electrons, and thus change the effective refractive index, finally leading to drastic color change. Recently, LCs have been integrated onto the plasmonic structures for pixelized color display by coupling the plasmonic resonance with birefringence, resulting in wide color tuning. By altering the refractive index of LC, the grating-coupled propagating surface plasmon modes can be modulated (Figure 3E) (Franklin et al., 2015). Recently, commercially available highly birefringent LC (LCM1107, nematic LCs) has been used for the tunable photonics with n_o_ = 1.55 and n_e_ = 1.97, where n_o_ and n_e_ mean ordinary and extraordinary refractive index. By applying the voltage, the orientation of LC molecules changed, causing surface plasmon resonance shift. Finally, the reflection spectra showed the resonance shifts as large as 95 nm with dynamic color change.

### Adaptive coloration for camouflage function

Squid, octopus, and other cephalopods show a capability for visibly adapting to their surrounding environment by changing color and texture, *i.e.*, camouflage. Inspired from the adaptive function, several researches including texturing manipulation (Xu et al., 2020) and pattern modulation (Yu et al., 2014) have been suggested. Along with previous studies, artificial camouflage using adaptive coloration is becoming an important subject for recently evolving fields such as soft robotics and electronic skin (Byun et al., 2018; Laschi et al., 2016). As shown in Figures 3F and 3A, color-tunable mechanical chameleon presented a real-time color change with matching background color (Wang et al., 2016a). The electrochemical deposition/strip of Ag shell layer surrounding Au nanostructures makes different plasmonic mode, showing a distinct color change. Furthermore, previous researches emphasized that not only color matching between background and object but also various disruptive patterns are important for effective camouflage (Stevens and Merilaita, 2009). Furthermore, the expression of disruptive patterns was presented through the thermochromic liquid crystals (TLC) based on the Ag nanowire (NW) heater (Kim et al., 2021b). When the current is applied to the NWs, Joule heating occurred thus the cholesteric pitch of TLC is changed, resulting in color manipulation. By superposition of the temperature profiles from the vertically stacked NW heaters, the chameleon model successfully showed fine patterns, improving the camouflage characteristics. Even if such materials show the great reversibility and color tunability, the full-color displays with high resolution still remains as a challenge. As M. L. Brongersma group presented futuristic display with 10000 pixels per inch (PPI) based on the meta-mirrors (Joo et al., 2020), the ultrahigh resolution colored display becomes feasible. Recently, colored display with etalon structure based on the swelling materials shows advanced resolution (∼12,000 PPI); however, pixel-by-pixel control with fast response is still limited (Jung et al., 2022). Therefore, the active materials, which could be scaled down (*e.g.*, PCM, conducting polymers), and addressable circuit are required for real-world applications.

## Active metasurface for flat optics

### Spatiotemporal metasurface and dynamic function

**Figure 4:**
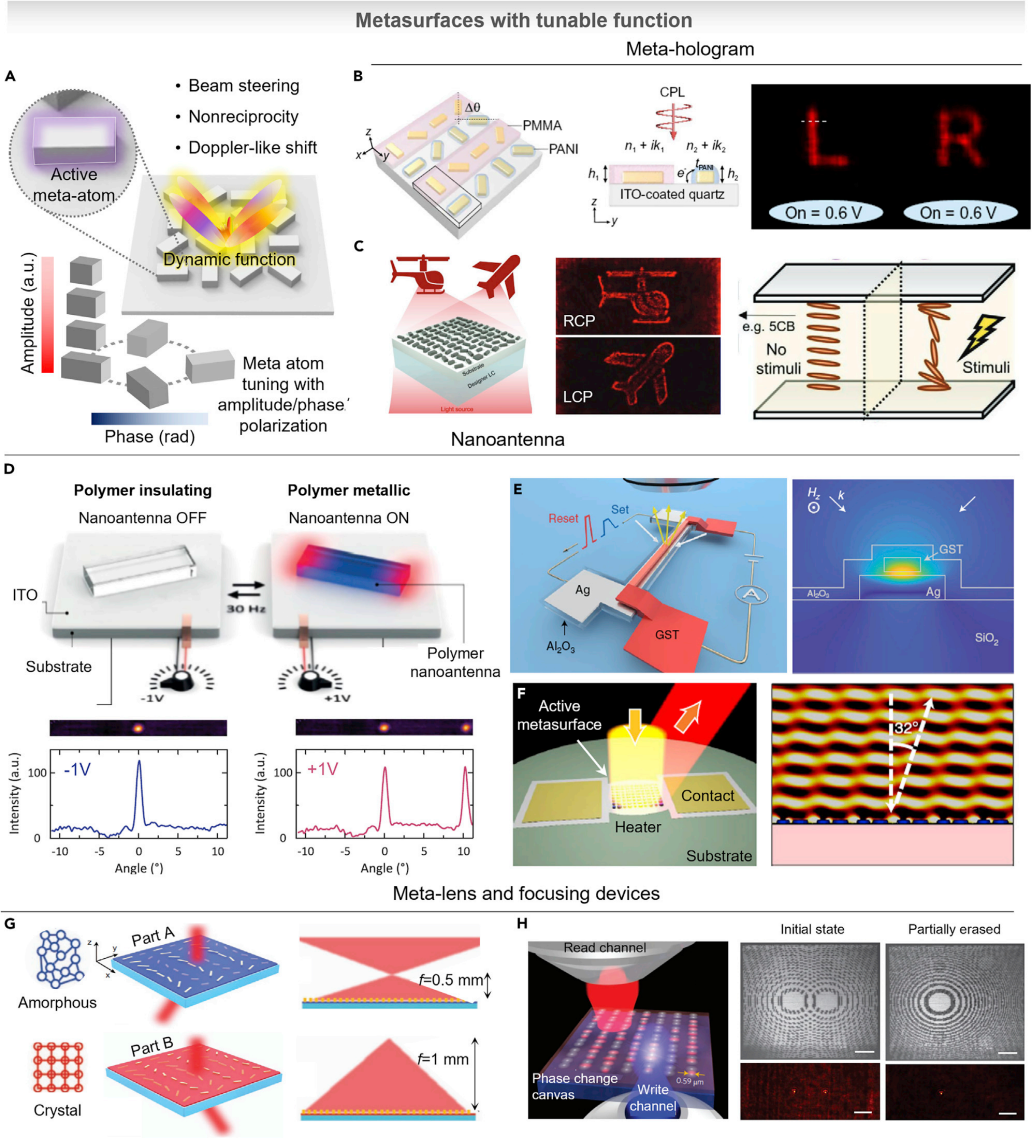
Metasurfaces for flat optics with tunable function (A) Schematic of dynamic metasurface based on active meta-atom with the function of tuning amplitude, phase, and polarization. (B) Schematic of meta-hologram based on metasurface coated with PANI for phase tuning. Reproduced with permission from (Kaissner et al., 2021), Copyright@2021 AAAS. (C) Meta-hologram based on LCs by controlling the polarization state of incident light (*i.e.*, tuning between left and right circularly polarized light). Reproduced with permission from (Kim et al., 2020a), Copyright@2020 John Wiley and Sons. (D) Beam steering based on PEDOT:PSS nanoantenna using the optical constant tunable property by applying electric potential. Reproduced with permission from (Karst et al., 2021), Copyright@2021 AAAS. (E) GST-326-based reversibly tunable nanoantenna by designing the structures on a small area of heating electrode for fast phase change. Reproduced with permission from (Wang et al., 2021b), Copyright@2021 Springer Nature. (F) Active metasurface based on large switching volume ability of GSST-2241 meta-atoms. Reproduced with permission from (Zhang et al., 2021), Copyright@2021 Springer Nature. (G) Tunable meta-lens based on GST-326 with the tunability of focal length. Reproduced with permission from (Yin et al., 2017). Copyright@2017 Springer Nature. (H) Reconfigurable metasurface based on light-induced phase change of GST-225 and dynamically tuning the functionality of the phase-change metasurface with SEM image. The scale bar is 10 μm. Reproduced with permission from (Wang et al., 2016b). Copyright@2015 Springer Nature.

Metasurfaces for flat optics have shown new approaches to control the light within flat and ultrathin optical components, which make local changes to the amplitude, phase, and polarization state of light. Thanks to the advantage of metasurface, this tiny optical system can implement many tasks which can be achieved by bulky optical components such as lens, hologram, and wavefront engineering. Nowadays, many progress in metasurface devices were presented with evolutionary development from passive to active (Krasnok, 2020; Lepeshov and Krasnok, 2021; Shaltout et al., 2019). The advantage of active metasurfaces is useful for the many applications that could provide benefits from having tunable optical components with flat/tiny, which make it easy to integrate. In accordance with optically active change of meta-atom, photons are spatiotemporally interacting with active metasurface. From the modulation, the frequency, linear and angular momentum, and spin can be controlled. As described in Figure 4A, these phenomena are clarified as operating principles, which is a light interaction of Doppler shifts, break Lorentz reciprocity, or produce time-reversed optical beams. Doppler-like wavelength shift is related to the time refraction (Shaltout et al., 2019). A photon propagating through the time-dependent variability of the refractive index can shift the frequency over time (Mendonça, 2000). In the nonreciprocal time-varying metasurface, the incident light can undergo a wavelength shift and reflect with a time-reversed beam causing a deviation of Snell’s law (Fang et al., 2012; Shi and Fan, 2016). In this section, we summarize the different types of metasurfaces and following applications including meta-hologram, nanoantenna, and meta-lens.

### Meta-hologram with switchable function

Holography is a promising imaging technology to reconstruct the three-dimensional image of object by recording the amplitude and phase information of light (Gabor, 1948). Owing to various applications in display, security, and data storage, holography has attracted attention. To achieve holographic images, spatial light modulators and diffractive optical elements are necessary. However, their low resolution has hindered the quality of hologram and subwavelength-scale modulator is required to achieve clear image without unnecessary diffraction orders obstructing the reconstructed holographic images (Lee et al., 2019). Replacing the conventional spatial light modulator, metasurface has emerged as an alternative platform to generate holograms. Although metasurface holograms offer extraordinary performances, the fixed meta-atoms take only static phase/amplitude information. In recent years, by combining optically active materials and metasurface, switchable/dynamic hologram shows a great tunability. Dynamic metasurface shows tunable hologram based on hierarchical reaction kinetics upon hydrogenation and dehydrogenation (Kaissner et al., 2021). Using the metallic meta-atoms of Mg/Pd, dynamic metasurface was constructed based on Pancharatnam-Berry (PB) phase and Au meta-atom was used as static metasurface. The two parts were interpolated into a set with a displacement vector of 300 nm, 300 nm (in vertical, lateral direction). From the static metasurface of Au, it always made an image of the Chinese word “Peace” by the incident light of right circularly polarized (RCP). In case of the meta-atom of Mg/Pd, the Chinese word “Harmony” was switched corresponding to the hydrogenation and dehydrogenation. In case of refractive index tuning by controlling redox state of conductive polymer, as shown in Figure 4B, PANI/PMMA-coated metasurface shows switchable holographic image with fast response time (35 ms) (Li et al., 2018a). In this study, meta-atoms were arranged based on PB phase, which has the pairs with angle difference (90°) between nanorods. As described in Figure 4B, the passive polymer capping layer of PMMA is covered on odd raw and the active layer of PANI is covered on even layer. At the leucoemeraldine state of PANI (reduced form), PANI has complex refractive indices (*n* = 1.5, *k* = 0), same as those of PMMA. In this case, from the incident light, the scattered light of neighboring rows has same intensity but shows phase difference in π between the light waves scattered by the odd and even rows, resulting in destructive interference with zero reflection. On the other hand, at the emeraldine state of PANI (oxidized form), its k increases while n decreases. In this case, PANI is highly absorptive; therefore, the scattered light from the PANI-conjugated rows is diminished. As a result, the odd row covered by PMMA acts as nanoantennas; finally, the reflected light switches with high intensity contrast. By tuning the polarization state of the incident light based on LCs, active hologram has been demonstrated. As described in Figure 4C, different holographic images are displayed depending on the circular polarization state of the incident light by designing meta-atoms to exploit both the propagation and geometric phase (Kim et al., 2020a). This operation is realized by the LC cell (a nematic LC, 4-Cyano-4′-pentylbiphenyl, 5CB). In normal state, the retardation of the phase of incident light produces left circularly polarized (LCP) light, on the other hand, in stimulated state, the incident light produces RCP phase.

### Active nanoantenna for wavefront modulation

As a cornerstone for developing a fast/reliable metasurface, wavefront steering is demonstrated using active nanoantenna based on newly applied active material and/or meta-atom structure. As described in Figure 4D, switchable nanoantenna is demonstrated based on the (poly(3,4-ethylenedioxythiophene):polystyrene sulfonate, PEDOT:PSS) with an electrochemically driven metal-to-insulator transition in the near-infrared spectral range (Karst et al., 2021). Caused by the charge carrier density variation, the refractive index is changed. Under the bias of +1 V, the PEDOT:PSS shows metallic optical property owing to its electrochemical doping state (oxidized) with high carrier concentration. In this state, nanoantennas showed a strong plasmonic resonance. On the other hand, under the bias of −1 V, the carrier is reduced, and polymer layer becomes insulating; consequently, nanoantennas are switched off. This electrochemical reaction showed a fast response time under video-rate frequency up to 30 Hz.

Meanwhile, PCMs-based chalcogenide alloys have attracted great attention due to their giant refractive index contrast with non-volatile properties. However, in case of GST, because crystallization and re-amorphousization require thermal energy to reach the glass transition and melting points, the reversible reaction is difficult to change the entire metasurface. Recently, new approaches have been introduced: 1) applying pulsed electric power on the designed circuit (for Joule heating), which is integrated structure of GST metasurface, and 2) modifying the composition ratio of the GST by doping other chalcogen groups such as GSST. Figure 4E shows GST-based nanoantennas with electrically switchable functions (Wang et al., 2021b). Using this structure, Y. Wang et al. presented fast-tunable and reversible active nanoantennas. The silver nanostrips can act as a nano-heater with antenna function. Based on this structure, the optical antennas offer strong scattering from visible to near-infrared spectral range with modulation of the reflectance by more than 4-fold. As another approach of tuning the composition ratio of chalcogenide PCM, Y. Zhang et al. showed a GSST-2241-based metasurface which increases switching volume of PCM and imparts important advantages of mitigation of optical losses (Figure 4F) (Zhang et al., 2021). Using the engineered material, the metasurface showed an on-chip scale reconfigurable beam steering, which shows great reversibility and fast switching time (∼5 μs for amorphization, ∼ 500 ms for crystallization).

### Varifocal and reconfigurable meta-lens

Optical lenses are critical component for tremendous technology including camera, microscope, mobile phone, and optical lithography. On the basis of this conventional design, convex or concave refractive lenses are widely used for various applications; however, the bulky shapes not only limit miniaturization but also require time-consuming surface machining. Although diffractive lenses provide solution to realize thin/compact lens, the shadowing effect limits the focusing capability (Moon et al., 2020). To overcome the limitations, ultrathin meta-lens has been introduced, which is completely flat and impart a spatially controlling phase profile through modulating the wavefront. By combining the optically active materials and the meta-lens, reconfigurable and varifocal lenses have been presented to produce an advanced optical component for biosensing, optical imaging, and optical characterization. Figure 4G shows a reconfigurable GST-based bifocal zoom lens (Yin et al., 2017). Corresponding to the alternatively placed meta-atoms with two parts (part A and B), when the active layer is in the amorphous phase, only part A rods interact with the incident light and when the active layer is in the crystalline phase, only part B rods interact with the incident light at 3.1 μm wavelength. Consequently, the focal length shifts between 0.5 and 1 mm. This actively tunable meta-lens opens the opportunities for the focal length tunable flat lens without change of optical component. Another example shows a writable focusing device (meta-lens) based on optically reconfigurable metasurface (Figure 4H) (Wang et al., 2016b). The femtosecond-laser writes the patterns by changing the phase of GST-225 to record grayscale patterns consisting of crystalline nanomarks with a diffraction limited resolution (0.59 μm). In this work, using the reconfigurable platform, meta-lenses are patterned with capability to produce two focal spots. By carefully controlling the pulsed femtosecond laser, the recorded pattern was erased, and it showed only one focal spot. This write/erasable platform also presented hologram and resonant metasurfaces. Even though the PCMs offer high tunability and reversibility, they cause not only high-power consumption but also optical loss in visible, resulting in a barrier to applications on colored hologram and/or meta-lens. Therefore, there are many efforts to reduce the optical loss by engineering the material composition of the PCMs or using alternative active materials (*e.g.*, conducting-polymers-/LCs-based dynamic photonics).

## Photonic memory for integrated device

### Structures of photonic memory and operating mechanisms

**Figure 5:**
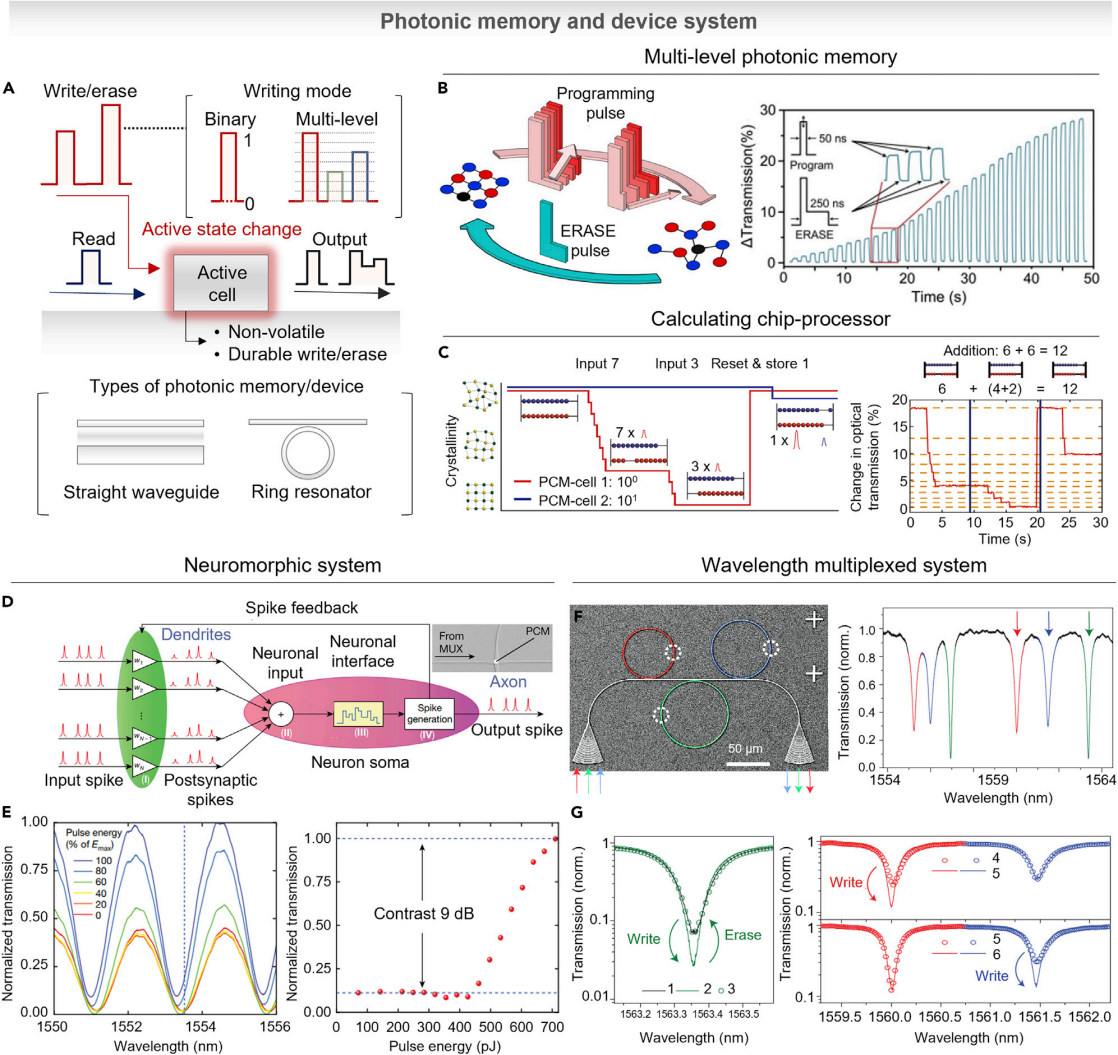
Photonic memory and device system (A) Schematic of ultrashort optical pulse to change the phase of active layer. By applying different power to the pulses, the information can write and erase information. There are several types of photonic structures including waveguide, ring resonator, and nanogap. (B) Illustration of the photonic memory cell with pulse shape and corresponding multilevel transmittance change. Reproduced with permission from (Li et al., 2019), Copyright@2019 The Optical Society. (C) A calculating chip-processor that shows optical abacus ability by applying multiple pulses to PCM cells. Reproduced with permission from (Feldmann et al., 2017), Copyright@2017 Springer Nature. (D) Neuronal circuit system consisting of pre-synaptic input and one post-synaptic output connected via PCM synapse. Reproduced with permission from (Feldmann et al., 2019), Copyright@2019 Springer Nature. (E) Normalized transmission measurement of the PCM synapse with change of coupling between ring and waveguide. Reproduced with permission from (Feldmann et al., 2019), Copyright@2019 Springer Nature. (F) Multi-bit, multi-wavelength non-volatile photonic memory enabled by GST-225 based waveguide with different resonating structures. Reproduced with permission from (Ríos et al., 2015), Copyright@2015 Springer Nature. (G) Multi-wavelength write/erase cycling with wavelength selective readout of individual cells. Reproduced with permission from (Ríos et al., 2015), Copyright@2015 Springer Nature.

The current issues for overcoming the limitation of data processing and transfer are the speed of information transfer. Over decades, the traditional approaches of chalcogenides and PCMs used in optical fiber, recently, on-chip photonics have been progressed by the development of nano-/micro-fabrication techniques (Cheng et al., 2017; Rios et al., 2014). The information transfer based on the photonic structure is highly desired because of their almost unlimited bandwidth and the ability of multiplexing. Over the bottleneck between information memory and processor in the so-called Neumann computer, the increased bandwidth of optical circuit is desired as a solution (Cheng et al., 2018; Wuttig et al., 2017). The combining PCMs with optical circuits have shown a capability for the ultrafast and low-loss functionality with non-volatile property (Ríos et al., 2019; Wright et al., 2019). As shown in the Figure 5A, the common types of integrated systems comprising photonic structures and active material show rewritable and non-volatile properties with capability of multilevel operations. In this section, we will introduce the recent progress of photonic memory devices and their applications.

### Multilevel photonic memory and processor

Recent advances in photonic structures and PCM have led to the development of photonic memory devices for high-capacity data storage, such as ferroelectric, resistive metal oxides and other promising applications such as magnetic random-access memories. Among these materials, GST is particularly attractive due to their high resistance contrast between the crystalline and amorphous state, which enables multilevel operation by controlling the ratio of the two states. Representatively, Figure 5B shows a schematic of programming pulse and erase pulse for the multilevel memory using GST-225 (Li et al., 2019). As described in the Figure 5B (inset), the high-power single pulse made an amorphous state (*i.e.*, write step), while a double pulse with two different power made crystalline state (*i.e.*, erase step). By controlling the amplitude and duration time of erase pulse, the degree of recrystallization is finely controlled. As a result, it presented 34 arbitrary levels, over 5 bits. The realization of multilevel photonic memory has a potential of arithmetic processing over the data storage. Figure 5C shows an example demonstration, using a straight rib waveguide (Feldmann et al., 2017). The two phase-change cells are switched by optical pulse with multilevel states. The represented example shows the calculation (7 + 3 = 10). As represented in the inset of Figure 5C, the PCM-cell 1 represents 10^0^ and PCM-cell 2 represents 10^1^. Once the phase is changed with seven and three steps, the first cell is reset, including carryover to a second cell, indicating that the calculation result is ten. Likewise, the addition (6 + 6 = 12) was performed with same process. As a potential of integrated phase-change photonics, Figure 5D represents the ability to provide all optical hardware by mimicking brain synapses and neurons (Feldmann et al., 2019). The represented neuron comprises N input (pre-synaptic) neurons, one output (post-synaptic) neuron, and N interconnecting synapses. Each connection has a certain weight, and the weighting is achieved via PCMs cell integrated on top of the waveguides. In this study, the system mimicked the behavior of neurons in a synaptic connection using a micro-ring resonator combined with PCMs cells. When the PCM cell is in the amorphous state, the synaptic waveguide is highly transmissive and represents a strong connection between two neurons. On the other hand, in the crystalline state, most of the light is absorbed and it leads to a weak connection. Figure 5E shows a normalized transmission of a ring resonator corresponding to the pulse energy. Due to the reduced absorption in the PCM cell, the transmission was increased, and right-side graph shows the activation function to define the firing threshold value.

### Wavelength-multiplexed photonic device

As a further step by illustrating single cell access, multi-bit/multi-wavelength photonic memory devices have been presented. Based on the PCMs cells coupled evanescently to a waveguide, as described in Figure 5F, on-chip nanophotonic device allows integration and exploitation of optical signal processing techniques such as wavelength division multiplexed (WDM) approaches (Ríos et al., 2015). With the wavelength-filtering property, on-chip optical cavities consist of one waveguide embedding GST-225 elements with three ring resonators coupled to the waveguide as represented in scanning electron microscope (SEM) image. Corresponding to each colored ring in the SEM image, three distinct resonances are distinguished in transmission spectra in Figure 5F. By applying the optical pulse with different wavelength, each cell is selectively addressed. The left panel of Figure 5G shows the initial state and recovered state after one write/erase cycle representing reversibility. The right panel of Figure 5G clearly shows the individually addressed transmittance corresponding to different input wavelengths. Such a controlling ability in multilevel memory processing allows us to approach the next-generation fast computational platform. However, PCMs intrinsically demand high power consumption (*i.e.*, energy loss), so we believe that searching for non-volatile and low-power consumption active materials (such as optically variable ferroelectric materials) is a crucial next step toward real-world optical memory applications (Geler-Kremer et al., 2022).

## Thermal radiation with tunable photonics

### Thermal radiation effect and emissivity tuning

**Figure 6:**
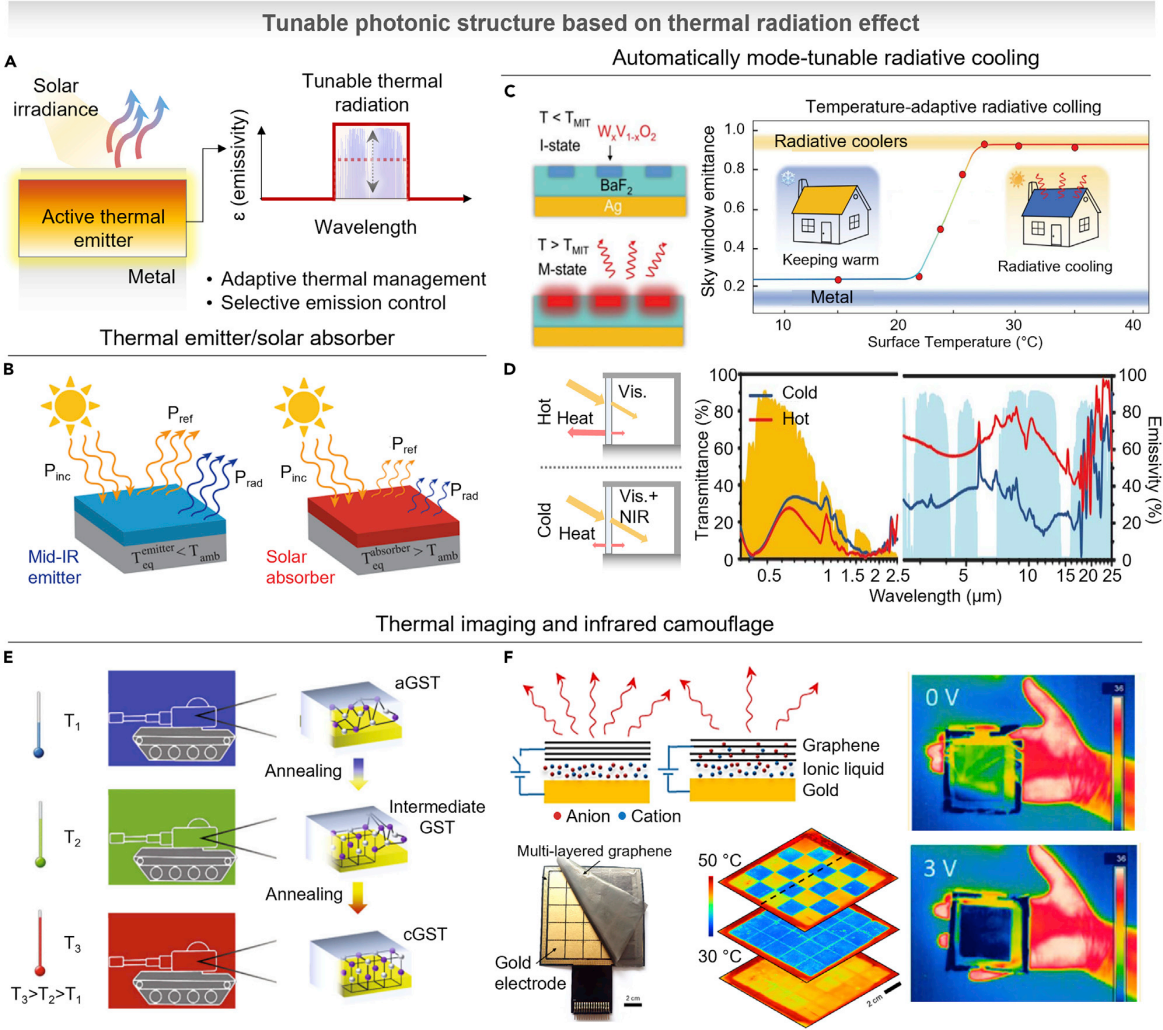
Tunable photonics based on thermal radiation effect (A) Schematic of tunable thermal radiation by selective emission control. In the graph, the purple-shaded area represents atmospheric transmittance. (B) Mode-tunable active photonic structure which modulates the function between thermal emitter and solar absorber. Reproduced with permission from (Kort-Kamp et al., 2018), Copyright@2018 American Chemical Society. (C) Temperature-adaptive radiative cooler based on W_x_V_1-x_O_2_ which actively changes the function between keeping warm and radiative cooling. Reproduced with permission from (Tang et al., 2021), Copyright@2021 AAAS. (D) Radiative cooling regulating thermochromic smart window based on VO_2_ which actively changes the function between keeping warm and radiative cooling. Reproduced with permission from (Wang et al., 2021a), Copyright@2021 AAAS. (E) Thermal camouflage for different background temperatures based on metal/GST-225 resonator. Reproduced with permission from (Qu et al., 2018), Copyright@2018 Springer Nature. (F) Thermal emission controllable graphene sheet by intercalating of ionic liquids, resulting in Fermi level variation. Reproduced with permission from (Salihoglu et al., 2018), Copyright@2018 American Chemical Society.

As one of several modes of heat transfer, thermal radiation exists in nature. Thermal radiation has a key important role in a wide range of technologies including thermal camouflage (Han et al., 2014), heat management (Raman et al., 2014), and imaging (Hulley et al., 2016). A representative example of thermal camouflage is infrared camouflage, which works to conceal infrared signals from targets and the environment. By matching the thermal radiation of object with target background, it hides the patterns from the infrared detector thus it has important role in modern systems for protecting vessels (Selzer and Gutfinger, 1989), aircraft (Mahulikar et al., 2007), vehicles (Ma et al., 2016), and humans (Chandrasekhar et al., 2002). Another example is heat management (*e.g.*, radiative cooling and solar energy absorption), which is a thermal energy emission into the universe. Around the temperature of ambient temperature (∼300 K), the atmospheric transmittance (highly transparent spectral range of Earth’s atmosphere, 8–13 μm) is matched with the black-body thermal radiation peak defined by Plank’s law (Hossain and Gu, 2016). According to the Planck’s law, any object having temperature above absolute zero emits thermal radiation, which is proportional to the surface emissivity (ε) and the fourth power of temperature (T). Therefore, to control the thermal radiation, precise control of emissivity is important. Recently, emissivity profile within atmospheric window is engineered for enhancing radiative cooling ability. Therefore, various progresses to improve the performance of cooling efficiency include thin films, scattering medium, and surface plasmonic structure (Heo et al., 2020; Kang et al., 2021; Kim et al., 2021a; Lee et al., 2018a). Recently, over the static thermal radiative photonics, the ability to control thermal emission offers a wide range of applications including tunable radiative cooling, thermophotovoltaics, and adaptive camouflage. As represented in Figure 6A, optically active materials integrated on thermal radiative photonics provide a tunable thermal emissivity, enabling dynamically mode changeable function. In this section, we will introduce the recent approaches to control the thermal radiation and its application.

### Mode-tunable photonics for heat management

First of all, over the static radiative cooler, Figure 6B shows a mode-tunable thermal management film (Kort-Kamp et al., 2018). Using the thermochromic phase-change materials (VO_2_), the multilayered structure presented radiative thermostats which act as radiative cooler at temperatures above the phase transition (>68°C), and solar absorbing heater at temperatures below the phase transition (<68°C). As described in Figure 6C, recently, Tang et al. experimentally presented a temperature-adaptive radiative coating for all-season household thermal regulation, with lowered phase-transition temperature (Tang et al., 2021). By setting the composition x of W_x_V_1-x_O_2_ to 1.5%, the transition temperature is tailored to ∼22°C. In the insulating state of W_x_V_1-x_O_2_ under transition temperature, it becomes insulator-state with heating mode. In case of higher temperature than transition temperature, the absorption is amplified by metallic property, which shows radiative cooling mode. Based on PCM, another approach demonstrated a smart window (Wang et al., 2021a), applied as an energy efficient part in building with tunable emissivity in atmospheric window (Figure 6D). Using the structure of W-doped VO_2_-PMMA/spacer/low-E stack, scalable and solution-process-based thermal modulation film was presented to manage the dynamic energy demand in different climate zones. This smart window controls indoor temperatures by modulating both NIR transmittance and heat emissivity. In summer, the near-infrared is blocked, far-infrared is strongly emitted, and the visible light is transmitted. In winter, both near-infrared and visible light enter the room and heat radiation is suppressed.

### Thermal imaging and infrared camouflage

Thermal imaging and camouflage are necessary for the field of infrared tagging, anticounterfeiting, and optical securities. Thermal imaging distinguishes the object by comparing the radiation temperature of background and object. In this regard, by matching the radiative power, the object can be thermally camouflaged. Recent progress has extended emissivity control to dynamic photonic structures including graphene (Brar et al., 2015), doped zinc oxide (Coppens and Valentine, 2017), and thermochromic materials (Liu and Padilla, 2016). Recently, the actively tunable properties have been extended to the thermal camouflage application with development of metamaterials based on transformation thermotics (Li et al., 2018b), using natural materials (Yang et al., 2015). In recent year, using the advent of optically tunable materials, Figure 6E represents the thermal camouflage device made of GST-225 coated on metal film (Qu et al., 2018). The device can hide the patterns at varying background temperature from 30°C to 50°C with wide observation angle from 0° to 60°. Furthermore, using the cavity-coupled plasmonic structure based on tunable VO_2_ layer, the cavity tuning is successfully demonstrated for thermal camouflage (Chandra et al., 2018). The plasmonic surface in semiconducting state shows distinct resonant over the spectral range, on the other hand, plasmonic surface in metallic state reflects entire incident light like a mirror. However, the discrete states of GST and VO_2_ in two or three level limit the applicability for matching the emissivity level into background conditions. By directly tuning the absorption itself, the thermal emission can be controlled, according to Kirchhoff’s law of thermal radiation (Kim et al., 2022). As indicated in Figure 6F, using ionic liquid and graphene gating, the absorptivity continuously modulates upon the charge density change of the graphene (Salihoglu et al., 2018). By intercalating the ionic liquids into graphene layers, the associated charge density increases and Fermi level shifts to higher energy, causing the suppression in the infrared absorption (Ergoktas et al., 2021). As represented in the thermal images, at 0 V, the high emissivity conveys the back-side thermal profiles; however, at 3 V, the suppressed emissivity screens the profiles. Based on these mechanisms, individually addressable infrared display with 5 × 5 sub-pixels was presented, showing temperature contrast of 10°C each pixel without crosstalk.

## Tunable photonics for terahertz plasmonic modulation

### Terahertz plasmonics and modulation principles

Terahertz electromagnetic wave lies on the leverage of both spectral regions (*i.e.*, between microwave and optical-wave frequencies), which enable innovative applications such as communications and sensing (Sizov and Rogalski, 2010; Yang and Lin, 2020). Also, over the tremendous developments in the millimeter wave band for 5G communication system, THz band is expected to achieve 6G communication (*i.e.*, from Gbps to Tbps level), giving the abundant bandwidth, low latency, and improved data transfer rate (Elayan et al., 2019; Rappaport et al., 2019). Furthermore, THz sensors have attracted much attention because of their non-destructive properties as well as being able to observe the intramolecular and intermolecular vibrational modes of many chemical and biological macromolecules in this region (Xu et al., 2022). However, due to the limitations of electronic and optical devices in this region, known as the THz gap, it was difficult to efficiently switch between electromagnetic waves and electrical power, and there is a lack of related functional devices to fill this technology gap.

**Figure 7:**
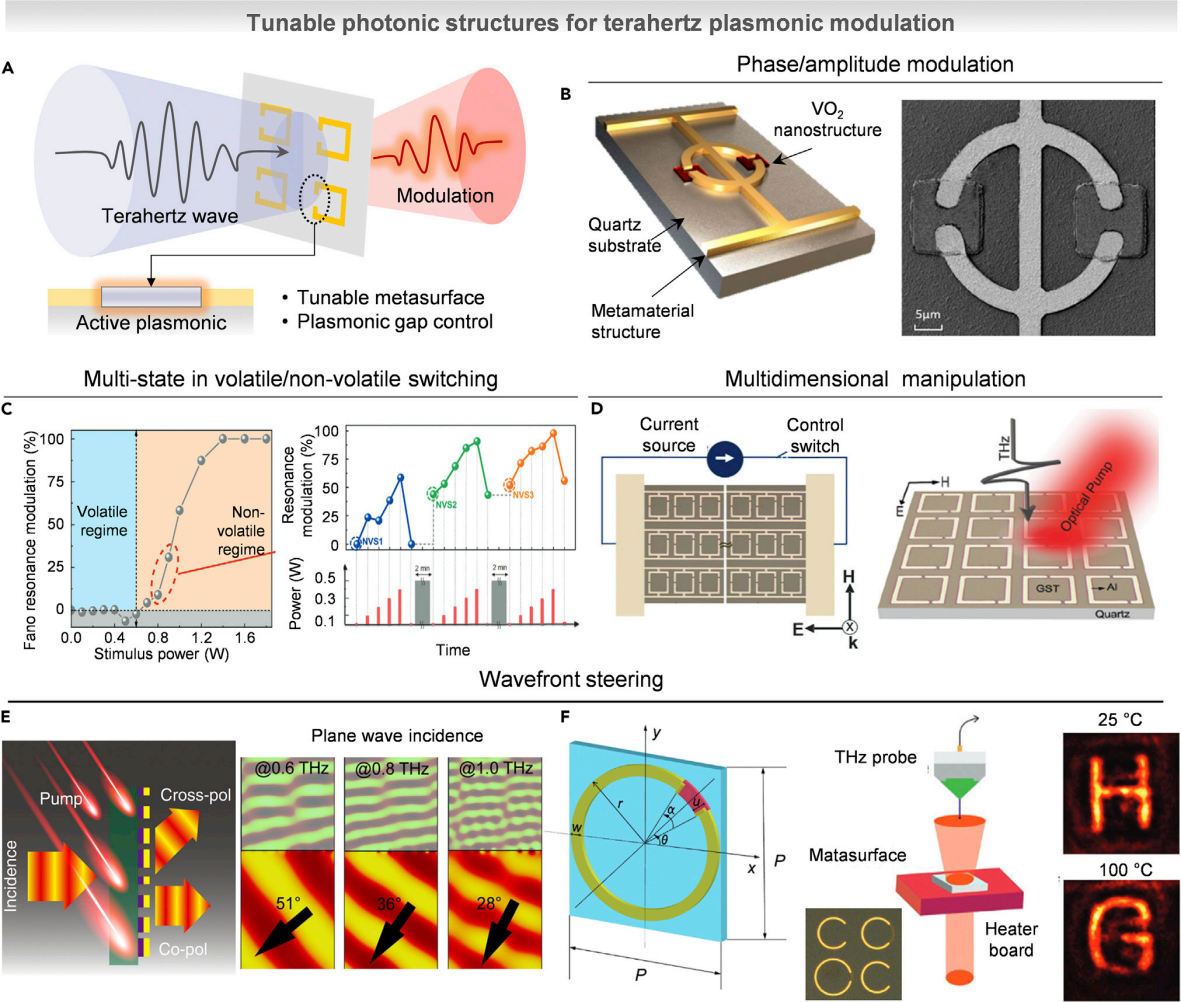
Tunable photonics for terahertz plasmonic modulation (A) Schematic of tunable terahertz plasmonic structure. (B) Phase/amplitude modulator based on ring-dumbbell composite resonator using VO_2_ (left) and SEM image (right). Reproduced with permission from (Zhao et al., 2018), Copyright@2018 American Chemical Society. (C) Volatile and non-volatile state tunable meta-device based on GST-225 (left) and resonance modulation corresponding to different pumping power (right). Reproduced with permission from (Pitchappa et al., 2021a), Copyright@2021 John Wiley and Sons. (D) Multidimensional manipulation based on thermally switchable multilevel nonvolatile states (left) and optically controllable ultrafast resonance switching (right). Reproduced with permission from (Pitchappa et al., 2019), Copyright@2019 John Wiley and Sons. (E) Terahertz dynamic beam splitter based on Si and Al hybrid structure using optical pumping. Reproduced with permission from (Cong et al., 2018), Copyright@2018 Springer Nature. (F) Active metasurface for terahertz hologram based on phase-transition material (VO_2_) by applying thermal energy. Reproduced with permission from (Liu et al., 2019b), Copyright@2019 John Wiley and Sons.

Because metasurface provides a strong confinement of incident electromagnetic field in a subwavelength region, it offers enhanced light-matter interaction, leading to the achievement of spectrally tunable filters, efficient polarizers, and beam steerers even in THz waves. In recent years, dynamic metasurfaces have shown efficient control of THz waves based on optically active material, liquid crystals, and MEMS technology. Among them, as described in Figure 7A, the integration of optically active materials into THz metadevices offers a selective modulation of the meta-atoms, enabling the realization of ultrafast applications. In this section, we introduced recent progress to realize the active tuning of metasurface including modulation of phase/amplitude, multi-state resonance, and wavefront manipulation.

### Phase and amplitude modulation in THz regime

Tunable modulators, which are capable of actively manipulating the phase and amplitude of THz waves, have attracted substantial attention because of their future applications in imaging, THz communication, and radar systems. However, most of materials exhibit only weak electrical and magnetic responses, limiting their ability to control THz radiation. Therefore, the introduction of metasurface enhanced the manipulation of electromagnetic radiation in the THz range (Cong et al., 2015). As an example, Figure 7B shows the dynamic light-guided control of the phase and amplitude of terahertz waves using VO2-coupled nanostructures. (Zhao et al., 2018). The dipole resonance and capacitive-inductance resonance of the ring-dumbbell complex resonator are transformed by the photoinduced phase transition of VO_2_, resulting in a large phase shift. The SEM images showed a structure of ring-dumbbell composite resonator. As a functional approach for implementing both volatile and non-volatile switching of Fano resonance, Figure 7C presents a GST-225-integrated metasurface (Pitchappa et al., 2021a). Light stimulation below the threshold results in an ultrafast volatile response of GST-225, which alters the Fano resonance state. On the other hand, under optical stimuli above the threshold, a non-volatile state was achieved by multistep tunability. Furthermore, from the subthreshold optical stimulus, ultrafast volatile switching altered the Fano resonance (<40 ps). As a multidimensional manipulation of THz waves, GST-225-based metasurface presented a Fano state change using optical and electrothermal stimulation. As represented in the left panel of Figure 7D, the metasurface can be actively controlled by electrical stimulus to apply Joule heating, enabling Fano state change (Pitchappa et al., 2019). Furthermore, the right panel shows ultrafast resonance switching induced by optical pulse based on the pumping of the photo-excited free carriers, which increase the film conductivity with fast response time (∼GHz of tunable modulation speed). The demonstration of manipulation by two different input parameters (thermal-induced phase change and optical pumping) realized the multifunctional terahertz metadevice.

### Wavefront modulation and holography

Wavefront modulation of THz wave plays a role for the applications such as beam splitting, beam focusing to manipulate the orbital angular momentum in a vortex beam, which carry information with improving the communication capacity, and holographic imaging for dynamic display (Kildishev et al., 2013; Yu et al., 2011). Figure 7E shows a beam steering and splitting of Si/Al hybrid circular split-ring resonator controlled by an external optical pump (Cong et al., 2018). By excitation of photocarriers, the resonance mode was switched to on state with the ultrafast switching time (∼667 ps). Finally, the active beam splitting was successfully demonstrated in a broadband range from 0.6 to 1.0 THz. As a functional application, dynamic terahertz meta-hologram has been demonstrated using thermally controllable metasurface using VO_2_. As represented in Figure 7F, the C-shaped split-ring resonators were used as a tunable metasurface, owing to their strong symmetric and anti-symmetric resonances, which show strong polarization anisotropy with broadband frequency (Liu et al., 2019b). Based on the phase transition of VO_2_ around 67°C, the metasurface generated different images at a static position.

## Summary and outlooks

In this review, we have summarized the recent advances of tunable photonics for efficiently manipulating electromagnetic waves from visible to THz spectral range, in conjunction with optically active materials. The progress of field has been rapidly evolving in the past decades providing plenty of promising achievements. Tunable photonics offer a potential route toward active control in spectral shape, amplitude, and phase of electromagnetic waves corresponding to each operation mechanism. From this development, highly beneficial applications have been experimentally demonstrated including color display, metasurfaces for flat optics, photonic memory, thermal radiation, and terahertz photonics. Nevertheless, tunable photonics are in their early stages of development and still have to mature with much more effort to meet the industrial needs. In the following, we discuss and outlook several aspects that should be required and considered in tunable photonics for future applications.

Tunable photonic structures with individual pixel-level control, such as meta-atoms or unit structures in the metasurfaces, should be implemented to be fully reconfigurable over a broad wavelength range. These initial attempts have been successfully demonstrated in applications with the control in unit structures of the metasurface with relatively large dimensions, specifically in the THz region using phase-change materials (Lin et al., 2022; Pitchappa et al., 2021b). In contrast, the fully reprogrammable unit structures of tunable photonics in visible are inherently difficult to fabricate for experiments. Because in this wavelength region, nanoscale dimensions of unit structures that require highly focused and precise control of lasers for optical tuning are essential, and functional diodes with similar dimensions are also required for electrical tuning. Nevertheless, in the visible region, pixel-level control has been reported by using a spatial light modulator to control the phase of the incident light and tune the output projected image planes instead of individually controlling the unit structure for metaholographic applications (Qu et al., 2020). Manipulating individual unit structures to create fully tunable photonics at visible wavelengths is a highly sought-after commodity, but the limitations of realizing such nanoscale circuits prove to be insurmountable obstacles. To address this, techniques to miniaturize the necessary modulation systems or the discovery of new functional materials with great contrast of optical properties at visible wavelengths are avenues to explore.

In realizing diverse applications of tunable photonics, characteristic factors including driving conditions, energy consumption, optical loss, incident light state, and reversibility should be carefully considered according to the target application. Some tunable photonic structures based on PCMs, semiconductors, and graphene perform faster modulation rates even when reaching GHz frequencies and wider amplitude/phase tuning ranges, but may entail significant energy consumption. These concerns also exist in tunable photonic structures based on optical modulation using laser pumping, even when modulation rates can reach the picosecond or femtosecond level. Furthermore, an absorption issue in short wavelength around visible frequency fundamentally limits the range of many optical applications (Zhang et al., 2019). On the other hand, in electrochemical modulation, the chemical reaction limits the modulation rate, but the requirements for external driving conditions are relatively mild and reversible with transparent characteristics even at short wavelength range (*e.g.*, PANI, visible range). In addition, electrochemical methods for controlling various photonic structures play an essential role in accelerating chemical reactions. Therefore, these electrochemical methods are widely presented in the design of functional devices based on tunable photonic structures with very low power consumption, even lower than that of commercial e-papers (Neubrech et al., 2020). In addition to the optical response tuning with material itself, the modulating ability also vary depending on the external control inputs. For example, the structural reconfiguration is also an effective strategy to achieve tunable photonic structures. The mechanical deformation and shape memory effect permit the optical tuning, but the difficulty of material synthesis, complicated fabrication process, and high cost are still significant problems to be solved (Che et al., 2020). In addition, the incident light conditions provide different optical responses, such as, polarization state (Jang et al., 2019; Kim et al., 2018; Lee et al., 2018b), orbital angular momentum (Ren et al., 2020), spin conditions (Ansari et al., 2019, 2020), and coherent properties (Yoon et al., 2018) of the incident light.

In conclusion, with the rapid development of modern physics and manufacturing technology in recent years, optically active material-based dynamic photonics have shown a developed platform toward achieve practical/futuristic applications, while it still shows their limitations that should be overcome. Therefore, to satisfy all the industrial needs, *e.g.*, the fast response, cost-effective manufacturing, broadband operation frequency, addressable pixelation, and portable/miniaturized instrumentation, further technical advances are necessary. With the rapid development of nanofabrication technology, fundamental analysis of optical properties of materials, miniaturized light sources, and interconnection with smart devices, we believe that dynamic photonics can be applied into diverse multifunctional devices and provide revolutionary opportunities including industry, military, and even in daily life.

### Limitation of the study

Due to the enormous number of research and steadily growing reports in the field of tunable photonics and optically active materials, it is impossible to cover and cite all publications. It is pointed out that we have focused on providing applications for each wavelength (*i.e.*, its own characteristics) and suggested corresponding guidelines for the materials selection. Meanwhile, including the optically active materials mentioned in this paper, there are many excellent and promising materials for implementing dynamic photonic responses. In this regard, we inform that our study just aimed at presenting the representative materials with the applications on the targeted (but broad) wavelengths.

## References

[bib1] Abdollahramezani S., Hemmatyar O., Taghinejad H., Krasnok A., Kiarashinejad Y., Zandehshahvar M., Alù A., Adibi A. (2020). Tunable nanophotonics enabled by chalcogenide phase-change materials. Nanophotonics.

[bib2] Ansari M.A., Kim I., Lee D., Waseem M.H., Zubair M., Mahmood N., Badloe T., Yerci S., Tauqeer T., Mehmood M.Q., Rho J. (2019). A spin-encoded all-dielectric metahologram for visible light. Laser Photon. Rev..

[bib3] Ansari M.A., Kim I., Rukhlenko I.D., Zubair M., Yerci S., Tauqeer T., Mehmood M.Q., Rho J. (2020). Engineering spin and antiferromagnetic resonances to realize an efficient direction-multiplexed visible meta-hologram. Nanoscale Horiz..

[bib4] Bakan G., Ayas S., Saidzoda T., Celebi K., Dana A. (2016). Ultrathin phase-change coatings on metals for electrothermally tunable colors. Appl. Phys. Lett..

[bib5] Barbero C., Kötz R. (1994). Nanoscale dimensional changes and optical properties of polyaniline measured by in situ spectroscopic ellipsometry. J. Electrochem. Soc..

[bib6] Brar V.W., Sherrott M.C., Jang M.S., Kim S., Kim L., Choi M., Sweatlock L.A., Atwater H.A. (2015). Electronic modulation of infrared radiation in graphene plasmonic resonators. Nat. Commun..

[bib7] Byun J., Lee Y., Yoon J., Lee B., Oh E., Chung S., Lee T., Cho K.-J., Kim J., Hong Y. (2018). Electronic skins for soft, compact, reversible assembly of wirelessly activated fully soft robots. Sci. Robot..

[bib8] Cai H., Huang Q., Hu X., Liu Y., Fu Z., Zhao Y., He H., Lu Y. (2018). All-optical and ultrafast tuning of terahertz plasmonic metasurfaces. Adv. Opt. Mater..

[bib9] Chandra S., Franklin D., Cozart J., Safaei A., Chanda D. (2018). Adaptive multispectral infrared camouflage. ACS Photonics.

[bib164] Chandrasekhar P., Zay B.J., Birur G.C., Rawal S., Pierson E.A., Kauder L., Swanson T. (2022). Large, switchable electrochromism in the visible through far-infrared in conducting polymer devices. Adv. Funct. Mater..

[bib10] Che Y., Wang X., Song Q., Zhu Y., Xiao S. (2020). Tunable optical metasurfaces enabled by multiple modulation mechanisms. Nanophotonics.

[bib11] Cheng Z., Ríos C., Pernice W.H.P., Wright C.D., Bhaskaran H. (2017). On-chip photonic synapse. Sci. Adv..

[bib12] Cheng Z., Ríos C., Youngblood N., Wright C.D., Pernice W.H.P., Bhaskaran H. (2018). Device-level photonic memories and logic applications using phase-change materials. Adv. Mater..

[bib13] Chien C.-Y., Wu L.-Y., Sheu C.-R., Hsu C.-J., Huang C.-Y., Chen C.-H., Huang L.-Y., Lee S.-L., Tsai W.-C. (2017). Fast response time in liquid crystal cells doped with low concentrations of reactive mesogen via photopolymerization at low temperature. Opt. Mater. Express.

[bib14] Chung K., Yu S., Heo C.J., Shim J.W., Yang S.M., Han M.G., Lee H.S., Jin Y., Lee S.Y., Park N., Shin J.H. (2012). Flexible, angle-independent, structural color reflectors inspired by Morpho butterfly wings. Adv. Mater..

[bib15] Cong L., Srivastava Y.K., Zhang H., Zhang X., Han J., Singh R. (2018). All-optical active THz metasurfaces for ultrafast polarization switching and dynamic beam splitting. Light Sci. Appl..

[bib16] Cong L., Xu N., Han J., Zhang W., Singh R. (2015). A tunable dispersion-free terahertz metadevice with Pancharatnam–Berry-Phase-enabled modulation and polarization control. Adv. Mater..

[bib17] Coppens Z.J., Valentine J.G. (2017). Spatial and temporal modulation of thermal emission. Adv. Mater..

[bib18] Cui T., Bai B., Sun H. (2019). Tunable metasurfaces based on active materials. Adv. Funct. Mater..

[bib19] Delaney M., Zeimpekis I., Lawson D., Hewak D.W., Muskens O.L. (2020). A new family of ultralow loss reversible phase-change materials for photonic integrated circuits: Sb2S3 and Sb2Se3. Adv. Funct. Mater..

[bib20] Derkaoui I., Khenfouch M., Benkhali M., Rezzouk A. (2019).

[bib21] Dirac P.A.M. (1927). The quantum theory of the emission and absorption of radiation. Proc. R. Soc. Lond. Ser. A.

[bib22] Duan X., Kamin S., Sterl F., Giessen H., Liu N. (2016). Hydrogen-regulated chiral nanoplasmonics. Nano Lett..

[bib23] Eaves-Rathert J., Kovalik E., Ugwu C.F., Rogers B.R., Pint C.L., Valentine J.G. (2022). Dynamic color tuning with electrochemically actuated TiO2 metasurfaces. Nano Lett..

[bib24] Elayan H., Amin O., Shihada B., Shubair R.M., Alouini M.-S. (2019). Terahertz band: the last piece of RF spectrum puzzle for communication systems. IEEE Open J. Commun. Soc..

[bib25] Ergoktas M.S., Bakan G., Kovalska E., Le Fevre L.W., Fields R.P., Steiner P., Yu X., Salihoglu O., Balci S., Fal’ko V.I. (2021). Multispectral graphene-based electro-optical surfaces with reversible tunability from visible to microwave wavelengths. Nat. Photonics.

[bib26] Fang K., Yu Z., Fan S. (2012). Realizing effective magnetic field for photons by controlling the phase of dynamic modulation. Nat. Photonics.

[bib27] Fang X., Ren H., Gu M. (2020). Orbital angular momentum holography for high-security encryption. Nat. Photonics.

[bib28] Feigenbaum E., Diest K., Atwater H.A. (2010). Unity-order index change in transparent conducting oxides at visible frequencies. Nano Lett..

[bib29] Feldmann J., Stegmaier M., Gruhler N., Ríos C., Bhaskaran H., Wright C.D., Pernice W.H.P. (2017). Calculating with light using a chip-scale all-optical abacus. Nat. Commun..

[bib30] Feldmann J., Youngblood N., Wright C.D., Bhaskaran H., Pernice W.H.P. (2019). All-optical spiking neurosynaptic networks with self-learning capabilities. Nature.

[bib31] Fermi E. (1932). Quantum theory of radiation. Rev. Mod. Phys..

[bib32] Franklin D., Chen Y., Vazquez-Guardado A., Modak S., Boroumand J., Xu D., Wu S.-T., Chanda D. (2015). Polarization-independent actively tunable colour generation on imprinted plasmonic surfaces. Nat. Commun..

[bib33] Freestone I., Meeks N., Sax M., Higgitt C. (2007). The Lycurgus cup—a roman nanotechnology. Gold Bull..

[bib34] Fu F., Shang L., Chen Z., Yu Y., Zhao Y. (2018). Bioinspired living structural color hydrogels. Sci. Robot..

[bib35] Gabor D. (1948). A new microscopic principle. Nature.

[bib36] Gabor D. (1972). Holography, 1948-1971. Science.

[bib37] Geler-Kremer J., Eltes F., Stark P., Stark D., Caimi D., Siegwart H., Offrein B.J., Fompeyrine J., Abel S. (2022). A ferroelectric multilevel non-volatile photonic phase shifter. Nat. Photonics.

[bib38] Gu J., Singh R., Liu X., Zhang X., Ma Y., Zhang S., Maier S.A., Tian Z., Azad A.K., Chen H.-T. (2012). Active control of electromagnetically induced transparency analogue in terahertz metamaterials. Nat. Commun..

[bib39] Gu J., Wei H., Ren F., Guan H., Liang S., Geng C., Li L., Zhao J., Dou S., Li Y. (2022). VO2-Based infrared radiation regulator with excellent dynamic thermal management performance. ACS Appl. Mater. Interfaces.

[bib40] Han S., Cong L., Srivastava Y.K., Qiang B., Rybin M.V., Kumar A., Jain R., Lim W.X., Achanta V.G., Prabhu S.S. (2019). All-dielectric active terahertz photonics driven by bound states in the continuum. Adv. Mater..

[bib41] Han T., Bai X., Thong J.T.L., Li B., Qiu C.W. (2014). Full control and manipulation of heat signatures: cloaking, camouflage and thermal metamaterials. Adv. Mater..

[bib42] He Q., Youngblood N., Cheng Z., Miao X., Bhaskaran H. (2020). Dynamically tunable transmissive color filters using ultra-thin phase change materials. Opt. Express.

[bib43] Hempelmann J., Müller P.C., Ertural C., Dronskowski R. (2022). The orbital origins of chemical bonding in Ge− Sb− Te phase-change materials. Angew. Chem. Int. Ed. Engl..

[bib44] Heo S.-Y., Lee G.J., Kim D.H., Kim Y.J., Ishii S., Kim M.S., Seok T.J., Lee B.J., Lee H., Song Y.M. (2020). A Janus emitter for passive heat release from enclosures. Sci. Adv..

[bib45] Hossain M.M., Gu M. (2016). Radiative cooling: principles, progress, and potentials. Adv. Sci..

[bib46] Hosseini P., Wright C.D., Bhaskaran H. (2014). An optoelectronic framework enabled by low-dimensional phase-change films. Nature.

[bib47] Huang Y.-W., Lee H.W.H., Sokhoyan R., Pala R.A., Thyagarajan K., Han S., Tsai D.P., Atwater H.A. (2016). Gate-tunable conducting oxide metasurfaces. Nano Lett..

[bib48] Hulley G.C., Duren R.M., Hopkins F.M., Hook S.J., Vance N., Guillevic P., Johnson W.R., Eng B.T., Mihaly J.M., Jovanovic V.M. (2016). High spatial resolution imaging of methane and other trace gases with the airborne Hyperspectral Thermal Emission Spectrometer (HyTES). Atmos. Meas. Tech..

[bib49] Jang J., Jeong H., Hu G., Qiu C., Nam K.T., Rho J. (2019). Kerker-conditioned dynamic cryptographic nanoprints. Adv. Opt. Mater..

[bib50] Jeong Y.G., Bahk Y., Kim D. (2020). Dynamic terahertz plasmonics enabled by phase-change materials. Adv. Opt. Mater..

[bib51] Joo W.-J., Kyoung J., Esfandyarpour M., Lee S.-H., Koo H., Song S., Kwon Y.-N., Song S.H., Bae J.C., Jo A. (2020). Metasurface-driven OLED displays beyond 10, 000 pixels per inch. Science.

[bib52] Julian M.N., Williams C., Borg S., Bartram S., Kim H.J. (2020). Reversible optical tuning of GeSbTe phase-change metasurface spectral filters for mid-wave infrared imaging. Optica.

[bib53] Jung C., Kim S.-J., Jang J., Ko J.H., Kim D., Ko B., Song Y.M., Hong S.H., Rho J. (2022). Disordered-nanoparticle-based etalon for ultrafast gas-responsive colorimetric sensors and anti-counterfeiting display. Sci. Adv..

[bib54] Kaissner R., Li J., Lu W., Li X., Neubrech F., Wang J., Liu N. (2021). Electrochemically controlled metasurfaces with high-contrast switching at visible frequencies. Sci. Adv..

[bib55] Kang M.H., Lee G.J., Lee J.H., Kim M.S., Yan Z., Jeong J.W., Jang K.I., Song Y.M. (2021). Outdoor-useable, wireless/battery-free patch-type tissue oximeter with radiative cooling. Adv. Sci..

[bib56] Karst J., Floess M., Ubl M., Dingler C., Malacrida C., Steinle T., Ludwigs S., Hentschel M., Giessen H. (2021). Electrically switchable metallic polymer nanoantennas. Science.

[bib57] Kildishev A.V., Boltasseva A., Shalaev V.M. (2013). Planar photonics with metasurfaces. Science.

[bib58] Kim M., Kim I., Jang J., Lee D., Nam K., Rho J. (2018). Active color control in a metasurface by polarization rotation. Appl. Sci..

[bib59] Kim D.H., Yoo Y.J., Ko J.H., Kim Y.J., Song Y.M. (2019). Standard red green blue (sRGB) color representation with a tailored dual-resonance mode in metal/dielectric stacks. Opt. Mater. Express.

[bib60] Kim Y.J., Yoo Y.J., Lee G.J., Yoo D.E., Lee D.W., Siva V., Song H., Kang I.S., Song Y.M. (2019). Enlarged color gamut representation enabled by transferable silicon nanowire arrays on metal–insulator–metal films. ACS Appl. Mater. Interfaces.

[bib61] Kim I., Ansari M.A., Mehmood M.Q., Kim W.S., Jang J., Zubair M., Kim Y.K., Rho J. (2020). Stimuli-responsive dynamic metaholographic displays with designer liquid crystal modulators. Adv. Mater..

[bib62] Kim I., Yun J., Badloe T., Park H., Seo T., Yang Y., Kim J., Chung Y., Rho J. (2020). Structural color switching with a doped indium-gallium-zinc-oxide semiconductor. Photonics Res..

[bib63] Kim Y.J., Yoo Y.J., Kang M.H., Ko J.H., Park M.R., Yoo D.E., Lee D.W., Kim K., Kang I.-S., Song Y.M. (2020). Mechanotunable optical filters based on stretchable silicon nanowire arrays. Nanophotonics.

[bib64] Kim D.H., Lee G.J., Heo S.-Y., Son S., Kang K.M., Lee H., Song Y.M. (2021). Ultra-thin and near-unity selective emitter for efficient cooling. Opt Express.

[bib65] Kim H., Choi J., Kim K.K., Won P., Hong S., Ko S.H. (2021). Biomimetic chameleon soft robot with artificial crypsis and disruptive coloration skin. Nat. Commun..

[bib66] Kim I., Martins R.J., Jang J., Badloe T., Khadir S., Jung H.-Y., Kim H., Kim J., Genevet P., Rho J. (2021). Nanophotonics for light detection and ranging technology. Nat. Nanotechnol..

[bib67] Kim Y., Kim C., Lee M. (2022). Parallel laser printing of a thermal emission pattern in a phase-change thin film cavity for infrared camouflage and security. Laser Photon. Rev..

[bib68] Ko B., Chae J.-Y., Badloe T., Kim H., Kim S.-J., Hong S.-H., Paik T., Rho J. (2022). Multilevel absorbers via the integration of undoped and tungsten-doped multilayered vanadium dioxide thin films. ACS Appl. Mater. Interfaces.

[bib69] Ko J.H., Yoo Y.J., Kim Y.J., Lee S., Song Y.M. (2020). Flexible, large-area covert polarization display based on ultrathin lossy nanocolumns on a metal film. Adv. Funct. Mater..

[bib70] Kort-Kamp W.J., Kramadhati S., Azad A.K., Reiten M.T., Dalvit D.A.R. (2018). Passive radiative “thermostat” enabled by phase-change photonic nanostructures. ACS Photonics.

[bib71] Krasnok A. (2020). Metalenses go atomically thick and tunable. Nat. Photonics.

[bib72] Laschi C., Mazzolai B., Cianchetti M. (2016). Soft robotics: technologies and systems pushing the boundaries of robot abilities. Sci. Robot..

[bib73] Lee G.J., Kim Y.J., Kim H.M., Yoo Y.J., Song Y.M. (2018). Colored, daytime radiative coolers with thin-film resonators for aesthetic purposes. Adv. Opt. Mater..

[bib74] Lee H.-E., Ahn H.-Y., Mun J., Lee Y.Y., Kim M., Cho N.H., Chang K., Kim W.S., Rho J., Nam K.T. (2018). Amino-acid-and peptide-directed synthesis of chiral plasmonic gold nanoparticles. Nature.

[bib75] Lee G.Y., Sung J., Lee B. (2019). Recent advances in metasurface hologram technologies. ETRI J..

[bib76] Lee J., Kim J., Lee M. (2020). High-purity reflective color filters based on thin film cavities embedded with an ultrathin Ge2Sb2Te5 absorption layer. Nanoscale Adv..

[bib77] Lee J.H., Kim Y.J., Yoo Y.J., Chang S., Lee G.J., Ko J.H., Kang K.M., Chanda D., Song Y.M. (2021). Colored, covert infrared display through hybrid planar-plasmonic cavities. Adv. Opt. Mater..

[bib78] Lee W., Yoo Y.J., Park J., Ko J.H., Kim Y.J., Yun H., Kim D.H., Song Y.M., Kim D.-H. (2022). Perovskite microcells fabricated using swelling-induced crack propagation for colored solar windows. Nat. Commun..

[bib79] Lepeshov S., Krasnok A. (2021). Tunable phase-change metasurfaces. Nat. Nanotechnol..

[bib80] Li Z., Clark A.W., Cooper J.M. (2016). Dual color plasmonic pixels create a polarization controlled nano color palette. ACS Nano.

[bib81] Li L., Jun Cui T., Ji W., Liu S., Ding J., Wan X., Bo Li Y., Jiang M., Qiu C.-W., Zhang S. (2017). Electromagnetic reprogrammable coding-metasurface holograms. Nat. Commun..

[bib82] Li J., Kamin S., Zheng G., Neubrech F., Zhang S., Liu N. (2018). Addressable metasurfaces for dynamic holography and optical information encryption. Sci. Adv..

[bib83] Li Y., Bai X., Yang T., Luo H., Qiu C.-W. (2018). Structured thermal surface for radiative camouflage. Nat. Commun..

[bib84] Li X., Youngblood N., Ríos C., Cheng Z., Wright C.D., Pernice W.H., Bhaskaran H. (2019). Fast and reliable storage using a 5 bit, nonvolatile photonic memory cell. Optica.

[bib85] Li H., Zhang X., Zhou F., Xiao X., Xu Y., Zhang Z. (2022). Tunable color gamut based a symmetric microcavity governed by Sb2S3. Opt Commun..

[bib86] Lin Q.W., Wong H., Huitema L., Crunteanu A. (2022). Coding metasurfaces with reconfiguration capabilities based on optical activation of phase-change materials for terahertz beam manipulations. Adv. Opt. Mater..

[bib87] Liu X., Padilla W.J. (2016). Thermochromic infrared metamaterials. Adv. Mater..

[bib88] Liu B., Liu J., Ji H., Wang W., Shen J., Zhang B. (2019). Terahertz nonvolatile in situ electrically erasable-rewritable photo-memory based on indium oxide/PEDOT: PSS. Opt Express.

[bib89] Liu X., Wang Q., Zhang X., Li H., Xu Q., Xu Y., Chen X., Li S., Liu M., Tian Z. (2019). Thermally dependent dynamic meta-holography using a vanadium dioxide integrated metasurface. Adv. Opt. Mater..

[bib90] Liu H., Dong W., Wang H., Lu L., Ruan Q., Tan Y.S., Simpson R.E., Yang J.K.W. (2020). Rewritable color nanoprints in antimony trisulfide films. Sci. Adv..

[bib163] Ma J., Chen C., Li C., Huang J. (2016). Infrared and visible image fusion via gradient transfer and total variation minimization. Inf. Fusion.

[bib91] Ma D., Wang J. (2017). Inorganic electrochromic materials based on tungsten oxide and nickel oxide nanostructures. Stem Cell Res..

[bib162] Mahulikar S.P., Sonawane H.R., Rao G.A. (2007). Infrared signature studies of aerospace vehicles. Prog. Aerosp. Sci..

[bib92] Makino K., Kato K., Saito Y., Fons P., Kolobov A.V., Tominaga J., Nakano T., Nakajima M. (2019). Terahertz spectroscopic characterization of Ge 2 Sb 2 Te 5 phase change materials for photonics applications. J. Mater. Chem. C Mater..

[bib93] Mendonça J.T. (2000).

[bib94] Mikheeva E., Kyrou C., Bentata F., Khadir S., Cueff S.b., Genevet P. (2022). Space and time modulations of light with metasurfaces: recent progress and future prospects. ACS Photonics.

[bib95] Moon S.-W., Kim Y., Yoon G., Rho J. (2020). Recent progress on ultrathin metalenses for flat optics. iScience.

[bib96] Neubrech F., Duan X., Liu N. (2020). Dynamic plasmonic color generation enabled by functional materials. Sci. Adv..

[bib97] Noginov M., Gu L., Livenere J., Zhu G., Pradhan A.K., Mundle R., Bahoura M., Barnakov Y.A., Podolskiy V.A. (2011). Transparent conductive oxides: plasmonic materials for telecom wavelengths. Appl. Phys. Lett..

[bib98] Ozbay E. (2006). Plasmonics: merging photonics and electronics at nanoscale dimensions. Science.

[bib99] Peng J., Jeong H.-H., Lin Q., Cormier S., Liang H.-L., De Volder M.F.L., Vignolini S., Baumberg J.J. (2019). Scalable electrochromic nanopixels using plasmonics. Sci. Adv..

[bib100] Peng J., Jeong H.H., Smith M., Chikkaraddy R., Lin Q., Liang H.L., De Volder M.F.L., Vignolini S., Kar-Narayan S., Baumberg J.J. (2021). FullyPrinted flexible plasmonic metafilms with directional color dynamics. Adv. Sci..

[bib101] Pitchappa P., Kumar A., Prakash S., Jani H., Venkatesan T., Singh R. (2019). Chalcogenide phase change material for active terahertz photonics. Adv. Mater..

[bib102] Pitchappa P., Kumar A., Prakash S., Jani H., Medwal R., Mishra M., Rawat R.S., Venkatesan T., Wang N., Singh R. (2021). Volatile ultrafast switching at multilevel nonvolatile states of phase change material for active flexible terahertz metadevices. Adv. Funct. Mater..

[bib103] Pitchappa P., Kumar A., Singh R., Lee C., Wang N. (2021). Terahertz MEMS metadevices. J. Micromech. Microeng..

[bib104] Qu G., Yang W., Song Q., Liu Y., Qiu C.-W., Han J., Tsai D.-P., Xiao S. (2020). Reprogrammable meta-hologram for optical encryption. Nat. Commun..

[bib105] Qu Y., Li Q., Cai L., Pan M., Ghosh P., Du K., Qiu M. (2018). Thermal camouflage based on the phase-changing material GST. Light Sci. Appl..

[bib106] Raman A.P., Anoma M.A., Zhu L., Rephaeli E., Fan S. (2014). Passive radiative cooling below ambient air temperature under direct sunlight. Nature.

[bib107] Rappaport T.S., Xing Y., Kanhere O., Ju S., Madanayake A., Mandal S., Alkhateeb A., Trichopoulos G.C. (2019). Wireless communications and applications above 100 GHz: opportunities and challenges for 6G and beyond. IEEE Access.

[bib108] Ren H., Fang X., Jang J., Bürger J., Rho J., Maier S.A. (2020). Complex-amplitude metasurface-based orbital angular momentum holography in momentum space. Nat. Nanotechnol..

[bib109] Ríos C., Hosseini P., Taylor R.A., Bhaskaran H. (2016). Color depth modulation and resolution in phase-change material nanodisplays. Adv. Mater..

[bib110] Rios C., Hosseini P., Wright C.D., Bhaskaran H., Pernice W.H.P. (2014). On-chip photonic memory elements employing phase-change materials. Adv. Mater..

[bib111] Ríos C., Stegmaier M., Hosseini P., Wang D., Scherer T., Wright C.D., Bhaskaran H., Pernice W.H.P. (2015). Integrated all-photonic non-volatile multi-level memory. Nat. Photon..

[bib112] Ríos C., Youngblood N., Cheng Z., Le Gallo M., Pernice W.H.P., Wright C.D., Sebastian A., Bhaskaran H. (2019). In-memory computing on a photonic platform. Sci. Adv..

[bib113] Rivera N., Kaminer I. (2020). Light–matter interactions with photonic quasiparticles. Nat. Rev. Phys..

[bib114] Rossi S., Olsson O., Chen S., Shanker R., Banerjee D., Dahlin A., Jonsson M.P. (2021). Dynamically tuneable reflective structural coloration with electroactive conducting polymer nanocavities. Adv. Mater..

[bib115] Rui G., Ding C., Gu B., Gan Q., Zhan Q., Cui Y. (2020). Symmetric Ge2Sb2Te5 based metamaterial absorber induced dynamic wide-gamut structural color. J. Opt..

[bib116] Salihoglu O., Uzlu H.B., Yakar O., Aas S., Balci O., Kakenov N., Balci S., Olcum S., Süzer S., Kocabas C. (2018). Graphene-based adaptive thermal camouflage. Nano Lett..

[bib117] Selvaraja S.K., Sethi P. (2018). Review on optical waveguides. Emerg. Waveguide Technol..

[bib161] Selzer F., Gutfinger D. (1989). LADAR and FLIR based sensor fusion for automatic target classification. SPIE.

[bib118] Shalaginov M.Y., An S., Zhang Y., Yang F., Su P., Liberman V., Chou J.B., Roberts C.M., Kang M., Rios C. (2021). Reconfigurable all-dielectric metalens with diffraction-limited performance. Nat. Commun..

[bib119] Shaltout A.M., Shalaev V.M., Brongersma M.L. (2019). Spatiotemporal light control with active metasurfaces. Science.

[bib120] Shastri B.J., Tait A.N., Ferreira de Lima T., Pernice W.H.P., Bhaskaran H., Wright C.D., Prucnal P.R. (2021). Photonics for artificial intelligence and neuromorphic computing. Nat. Photonics.

[bib121] Shcherbakov M.R., Liu S., Zubyuk V.V., Vaskin A., Vabishchevich P.P., Keeler G., Pertsch T., Dolgova T.V., Staude I., Brener I., Fedyanin A.A. (2017). Ultrafast all-optical tuning of direct-gap semiconductor metasurfaces. Nat. Commun..

[bib122] Shi Y., Fan S. (2016). Dynamic non-reciprocal meta-surfaces with arbitrary phase reconfigurability based on photonic transition in meta-atoms. Appl. Phys. Lett..

[bib123] Singh R., Azad A.K., Jia Q.X., Taylor A.J., Chen H.-T. (2011). Thermal tunability in terahertz metamaterials fabricated on strontium titanate single-crystal substrates. Opt. Lett..

[bib124] Sizov F., Rogalski A. (2010). THz detectors. Prog. Quant. Electron..

[bib125] Stevens M., Merilaita S. (2009). Defining disruptive coloration and distinguishing its functions. Philos. Trans. R. Soc. Lond. B Biol. Sci..

[bib126] Stevenson C.L., Bennett D.B., Lechuga-Ballesteros D. (2005). Pharmaceutical liquid crystals: the relevance of partially ordered systems. J. Pharm. Sci..

[bib127] Tang K., Dong K., Li J., Gordon M.P., Reichertz F.G., Kim H., Rho Y., Wang Q., Lin C.-Y., Grigoropoulos C.P. (2021). Temperature-adaptive radiative coating for all-season household thermal regulation. Science.

[bib128] Tian X., Li Z.-Y. (2018). An optically-triggered switchable mid-infrared perfect absorber based on phase-change material of vanadium dioxide. Plasmonics.

[bib129] Tong Z., Li N., Lv H., Tian Y., Qu H., Zhang X., Zhao J., Li Y. (2016). Annealing synthesis of coralline V2O5 nanorod architecture for multicolor energy-efficient electrochromic device. Sol. Energy Mater. Sol. Cell.

[bib130] Van Bilzen B., Homm P., Dillemans L., Su C.-Y., Menghini M., Sousa M., Marchiori C., Zhang L., Seo J.W., Locquet J.-P. (2015). Production of VO_2_ thin films through post-deposition annealing of V_2_O_3_ and VO_x_ films. Thin Solid Films.

[bib131] Wang G., Chen X., Liu S., Wong C., Chu S. (2016). Mechanical chameleon through dynamic real-time plasmonic tuning. ACS Nano.

[bib132] Wang Q., Rogers E.T.F., Gholipour B., Wang C.-M., Yuan G., Teng J., Zheludev N.I. (2016). Optically reconfigurable metasurfaces and photonic devices based on phase change materials. Nat. Photonics.

[bib133] Wang W., Ji H., Liu D., Xiong L., Hou Y., Zhang B., Shen J. (2018). Active bidirectional electrically-controlled terahertz device based on dimethyl sulfoxide-doped PEDOT: PSS. Opt Express.

[bib134] Wang K., Dahan R., Shentcis M., Kauffmann Y., Ben Hayun A., Reinhardt O., Tsesses S., Kaminer I. (2020). Coherent interaction between free electrons and a photonic cavity. Nature.

[bib135] Wang S., Jiang T., Meng Y., Yang R., Tan G., Long Y. (2021). Scalable thermochromic smart windows with passive radiative cooling regulation. Science.

[bib136] Wang Y., Landreman P., Schoen D., Okabe K., Marshall A., Celano U., Wong H.-S.P., Park J., Brongersma M.L. (2021). Electrical tuning of phase-change antennas and metasurfaces. Nat. Nanotechnol..

[bib137] Wright C.D., Bhaskaran H., Pernice W.H. (2019). Integrated phase-change photonic devices and systems. MRS Bull..

[bib138] Wu J.M., Liou L.B. (2011). Room temperature photo-induced phase transitions of VO2 nanodevices. J. Mater. Chem..

[bib139] Wuttig M., Bhaskaran H., Taubner T. (2017). Phase-change materials for non-volatile photonic applications. Nat. Photonics.

[bib140] Xu C., Colorado Escobar M., Gorodetsky A.A. (2020). Stretchable cephalopod-inspired multimodal camouflage systems. Adv. Mater..

[bib141] Xu C., Ren Z., Wei J., Lee C. (2022). Reconfigurable Terahertz Metamaterials: From Fundamental Principles to Advanced 6G Applications. iScience.

[bib142] Yang T., Bai X., Gao D., Wu L., Li B., Thong J.T.L., Qiu C.W. (2015). Invisible sensors: simultaneous sensing and camouflaging in multiphysical fields. Adv. Mater..

[bib143] Yang W., Lin Y.-S. (2020). Tunable metamaterial filter for optical communication in the terahertz frequency range. Opt Express.

[bib144] Yang Y., Yoon G., Park S., Namgung S.D., Badloe T., Nam K.T., Rho J. (2021). Revealing structural disorder in hydrogenated amorphous silicon for a low-loss photonic platform at visible frequencies. Adv. Mater..

[bib145] Yin X., Steinle T., Huang L., Taubner T., Wuttig M., Zentgraf T., Giessen H. (2017). Beam switching and bifocal zoom lensing using active plasmonic metasurfaces. Light Sci. Appl..

[bib146] Yoo Y.J., Kim W.G., Ko J.H., Kim Y.J., Lee Y., Stanciu S.G., Lee J.M., Kim S., Oh J.W., Song Y.M. (2020). Large-area virus coated ultrathin colorimetric sensors with a highly lossy resonant promoter for enhanced chromaticity. Adv. Sci..

[bib147] Yoo Y.J., Ko J.H., Kim W.-G., Kim Y.J., Kong D.-J., Kim S., Oh J.-W., Song Y.M. (2020). Dual-mode colorimetric sensor based on ultrathin resonating facilitator capable of nanometer-thick virus detection for environment monitoring. ACS Appl. Nano Mater..

[bib148] Yoo Y.J., Ko J.H., Lee G.J., Kang J., Kim M.S., Stanciu S.G., Jeong H.H., Kim D.H., Song Y.M. (2022). Gires–tournois immunoassay platform for label-free bright-field imaging and facile quantification of bioparticles. Adv. Mater..

[bib149] Yoon G., Lee D., Nam K.T., Rho J. (2018). “Crypto-display” in dual-mode metasurfaces by simultaneous control of phase and spectral responses. ACS Nano..

[bib150] Yu C., Li Y., Zhang X., Huang X., Malyarchuk V., Wang S., Shi Y., Gao L., Su Y., Zhang Y. (2014). Adaptive optoelectronic camouflage systems with designs inspired by cephalopod skins. Proc. Natl. Acad. Sci. USA.

[bib151] Yu N., Capasso F. (2014). Flat optics with designer metasurfaces. Proc. Natl. Acad. Sci. USA.

[bib152] Yu N., Genevet P., Kats M.A., Aieta F., Tetienne J.-P., Capasso F., Gaburro Z. (2011). Light propagation with phase discontinuities: generalized laws of reflection and refraction. Science.

[bib153] Zeng B., Huang Z., Singh A., Yao Y., Azad A.K., Mohite A.D., Taylor A.J., Smith D.R., Chen H.-T. (2018). Hybrid graphene metasurfaces for high-speed mid-infrared light modulation and single-pixel imaging. Light Sci. Appl..

[bib160] Zhai Y., Yang J.-Q., Zhou Y., Mao J.-Y., Ren Y., Roy V.A., Han S.-T. (2018). Toward non-volatile photonic memory: concept, material and design. Mater. Horiz..

[bib154] Zhang Y., Chou J.B., Li J., Li H., Du Q., Yadav A., Zhou S., Shalaginov M.Y., Fang Z., Zhong H. (2019). Broadband transparent optical phase change materials for high-performance nonvolatile photonics. Nat. Commun..

[bib155] Zhang Y., Fowler C., Liang J., Azhar B., Shalaginov M.Y., Deckoff-Jones S., An S., Chou J.B., Roberts C.M., Liberman V. (2021). Electrically reconfigurable non-volatile metasurface using low-loss optical phase-change material. Nat. Nanotechnol..

[bib156] Zhang Y.-n., Zhao Y., Lv R.-q. (2015). A review for optical sensors based on photonic crystal cavities. Sens. Actuator Phys..

[bib157] Zhao Q., Kang L., Du B., Li B., Zhou J., Tang H., Liang X., Zhang B. (2007). Electrically tunable negative permeability metamaterials based on nematic liquid crystals. Appl. Phys. Lett..

[bib158] Zhao Y., Zhang Y., Shi Q., Liang S., Huang W., Kou W., Yang Z. (2018). Dynamic photoinduced controlling of the large phase shift of terahertz waves via vanadium dioxide coupling nanostructures. ACS Photonics.

[bib159] Zheng J., Khanolkar A., Xu P., Colburn S., Deshmukh S., Myers J., Frantz J., Pop E., Hendrickson J., Doylend J. (2018). GST-on-silicon hybrid nanophotonic integrated circuits: a non-volatile quasi-continuously reprogrammable platform. Opt. Mater. Express.

